# Microscopic Analyses of Latent and Visible *Monilinia fructicola* Infections in Nectarines

**DOI:** 10.1371/journal.pone.0160675

**Published:** 2016-08-05

**Authors:** Carlos Garcia-Benitez, Paloma Melgarejo, Antonieta De Cal, Blanca Fontaniella

**Affiliations:** 1 Department of Plant Protection, Instituto Nacional de Investigación y Tecnología Agraria y Alimentaria (INIA), Madrid, Spain; 2 Department of Vegetal Biology I, Faculty of Biology, Complutense University of Madrid, Madrid, Spain; Fujian Agriculture and Forestry University, CHINA

## Abstract

Little is known about the histologic features of a latent *Monilinia fructicola* infection and brown rot in infected fruit. This report informs on the results of an investigation whose aim was to analyze the microanatomy of nectarines with a latent and visible *M*. *fructicola* infection. Mature nectarines were inoculated with an *M*. *fructicola* isolate and incubated at 25°C for 0, 24, 48, 72, or 96 hours in the dark. For investigating the latent infection process, the inoculated nectarines were first incubated at 25°C for 24 hours in the dark and then incubated at 4°C for 72, 144, 216, and 288 hours in the dark. At the end of the incubation, samples of nectarine tissue were excised from the inoculation points and prepared for light and transmission electron microscopic examinations. No signs of disease were seen on the surface of nectarines with a latent infection over the 288-hour incubation period. When the tissue samples were microscopically examined, *M*. *fructicola* colonized the stomata and this stomatal colonization progressively increased over time and was associated with gradual collapse of the epidermal cells and colonization of the subepidermis. In nectarines with visible brown rot, the disease usually appeared after 24 hours on the surface and in the uppermost layers of epidermal cells, which began to collapse after 48 hours. Subsequently, the diseased tissues of the nectarines displayed (a) colonization of the epidermis and mesocarp by *M*. *fructicola* with thin and thick hyphae, (b) collapse and disruption of epidermal and mesocarpic cells, (c) lysogenic cavities in the subepidermis and mesocarp, (d) degradation of the cuticle and epidermis, and (e) *M*. *fructicola* sporulation. *M*. *fructicola* is active during latent infections because slow and progressive colonization of nectarine subcuticular cells by the fungus occurs.

## Introduction

Brown rot is an economically important fungal disease of peaches (*Prunus persica* (L.) Batch) and nectarines (*P*. *persica* var. nectarina (Aiton) Maxim), and is responsible for substantial pre-harvest and post-harvest losses [1]. In Spain, brown rot is caused by three *Monilinia* spp.: *M*. *fructicola* (G Winter) Honey, *M*. *fructigena* (Aderhold & Ruhland) Honey, and *M*. *laxa* (Aderhold & Ruhland) Honey [2], and of these three fungi, *M*. *fructicola* has the fastest growth rate and is the most aggressive one [3]. Although fruit can be infected with *Monilinia* spp. at any stage of its development, disease incidence increases and the index of disease severity becomes greater with fruit ripening [4–6]. Brown rot on ripening or mature fruit typically develops as a rapidly spreading, firm, brown decay. Under optimum conditions, the decay of ripe infected peach and nectarine fruit may become visible within 48–72 hours of infection [2].

The fruit surface is a natural barrier against infection and must be penetrated before a pathogen can cause infection. For fungal infections of fruit, conidia must first be deposited on and adhere to the fruit surface before penetrating the surface through the natural openings and/or injured areas of the fruit surface [7,8]. For *Monilinia* spp., airborne conidia are first deposited on the fruit surface and penetration is implemented by germ tubes and/or appressoria [2] through stomata and/or natural cracks on fully mature (late stage III) fruit [9]. When the climatic conditions are unfavorable, *Monilinia* infections may remain latent until the conditions for disease development become favorable [2,10,11]. Latent infections have been described as a dynamic equilibrium between the host, pathogen, and environment without any visible signs of disease [5,12], or as an invisible infection that is chronic and in which a host and parasite relationship has been established [13]. Other authors differentiate between latent (non-visible) and dormant and quiescent (visible) infections in which a host-parasite relationship has been established [14]. Although a pathogen has a low metabolic level in a latent infection, pathogenicity factors may be activated to end the period of latent infection when conditions for disease development become conducive.

Fungal pathogens usually develop structures, such as appressoria, germ tubes, or hyphae, for penetrating and causing a latent infection in a host [15]. Intercellular hyphae have been described in a latent infection of (a) mangos with an *Alternaria alternate* infection [16], (b) mangos [17], avocados [18], citrus fruit [19], and blueberries [20] with a *Colletotrichum gloeosporioides* infection, and (c) soybeans [21] with a *Phialophora gregata* infection. Appressoria have been described in a latent infection of (a) unripe bananas [22] with a *C*. *musae* infection, (b) tangerines [19,23] and papayas [24] with a *C*. *gloeosporioides* infection, and (c) plums [25] with an *M*. *fructicola* infection. *Botrytis cinerea* uses an infection peg, which develops from a germinated appressorium to penetrate the nectarine or plum cuticle in a latent infection [26].

Latent infections due to *Monilinia spp*. have been detected in nectarines and plums [27,28], and a high correlation between post-harvest brown rot and latent infection exists in some stone fruit, such as peaches, plums, and cherries [5,6,12,29,30]. We have previously reported that the incidence of latent infection and that of post-harvest brown rot are positively correlated increasing both of them along the crop season: the average incidence of latent infection during the crop season in Spanish peach orchards explains 55% of the total variation in the incidence of post-harvest brown rot [5]. Gell et al. [5] have also developed a model of latent *Monilinia* infection in which temperature (T) and wetness duration (W) can be used for predicting disease outbreaks. Results of this multiple regression analysis indicate that T and W account for 83% of the total variation in the incidence of latent infection.

Little is known on the infection process during its transition from a latent *M*. *fructicola* infection to visible brown rot caused by *M*. *fructicola* in nectarines. Therefore, we conducted an investigation whose aim was to analyze the microanatomy of nectarines with a latent and visible *M*. *fructicola* infection using light and transmission electron microscopy.

## Materials and Methods

### Fungal cultures and conidial production

An isolate of *M*. *fructicola* (ALF 2009 COS 5R7), which was originally collected from a harvested nectarine in Alfarras, Lleida, Spain in 2009, was used in the investigation. The isolate was grown on potato dextrose agar (PDA) plates (Difco Laboratories, Detroit, MI, USA) at 20–25°C in the dark for seven days for conidial production. The isolate was stored either as a culture on PDA at 4°C for short-term storage or as a conidial suspension (see later for preparation) in 20% glycerol at −80°C for long-term storage.

Conidial suspensions were prepared using conidia that were harvested from the PDA plates by scratching the surface with a sterilized disposable scalpel after adding sterilized distilled water (SDW). The harvested conidia and mycelia were filtered through glass wool in order to remove the mycelia after a 30-second sonication in an ultrasonic bath (J.P. Selecta S.A., Barcelona, Spain). The filtrate was adjusted to the desired conidial concentration using SDW after counting the number of conidia using a hemocytometer and a light microscope (Zeiss Axioskop 2; Carl Zeiss, Inc., Oberkochem, Germany).

### Conidial germination on the nectarine´s epidermis

Samples of the epidermis (about 0.5 cm^2^ and 0.5–1 mm thick) of mature nectarines were obtained from surface-disinfected fruit using a sterilized disposable scalpel. To this end, the fruit surface was disinfected by first immersing the fruit in a 0.1% sodium hypochlorite solution for five minutes, followed by immersion in a 70% ethanol solution for one minute, and finally two rinses of the fruit in SDW. Samples of the epidermis were placed on the surface of 2% water agar plates (Laboratorios Conda S.A., Torrejón de Ardoz, Madrid, Spain). An aliquot (5 μl) of the conidial suspension (3x10^3^ conidia ml^-1^) was then deposited on each sample, and the samples were then incubated at 25°C or 4°C for four or 24 hours in the dark. At the end of the incubation, the samples were cleared by immersion in a 3:1 (v/v) mixture of ethanol and chloroform which contained 0.15% (w/v) trichloracetic acid for 15 minutes at 70°C, then immersed in a solution of phenol:water:glycerine:lactic acid (1:1:2:1 v/v) for two minutes, and then stained with lactophenol blue (Sigma-Aldrich Quimica SL., Madrid, Spain). After staining, each sample was mounted on glass slides in a solution of phenol:water:glycerine:lactic acid (1:1:2:1 v/v). Images of the specimens were examined after their capture by a Leica DFC550 camera, which was attached to a Leica DMRE microscope (Leica Microsystems, Wetzlar, Germany). Three samples were made for each temperature and incubation time. The controls were samples of the epidermis from uninoculated nectarines and the entire assay was repeated twice.

### Visible infection studies: fruit inoculation and incubation

Mature nectarines were used to investigate the visible infection process. For this purpose, the surface of the nectarines was first disinfected, as previously described, and then dried for two hours in a laminar flow cabinet. Once dried, four 30-μl drops of a conidial suspension (10^6^ conidia ml^-1^) were placed at different points on the surface close to the insertion point of the peduncle. The inoculated fruit were kept in the laminar flow cabinet until the drops evaporated. The fruit were then transferred to dry plastic plates in a humidity chamber at 100% relative humidity (RH) and incubated at 25°C for 0, 24, 48, 72, or 96 hours in the dark. One fruit was used for each incubation time. The controls were fruit which were inoculated with SDW and the entire assay was repeated twice.

### Latent infection studies: fruit inoculation and incubation

Mature nectarines were used to investigate the latent infection process. For this purpose, the surface of the nectarines was first disinfected, as previously described, and then dried for two hours in a laminar flow cabinet. Once dried, four 30-μl drops of a conidial suspension (10^6^ conidia ml^-1^) were placed at different points on the surface close to the insertion point of the peduncle. The inoculated fruit were then transferred to plates in polystyrene boxes which were lined with sterilized filter paper that was moistened with SDW and incubated at 25°C for 24 hours in the dark. After completion of the incubation, the surface of the inoculated nectarines was again disinfected, as previously described, and then dried for two hours in a laminar flow cabinet. Once dried, the fruit were transferred to dry plastic plates in a humidity chamber at 100% RH and incubated at 4°C for 72, 144, 216, and 288 hours in the dark. The controls were uninoculated fruit and the entire assay was repeated twice.

### Light microscopic analysis of nectarines with a visible or latent *M*. *fructicola* infection

At the end of the incubation, a sample of nectarine tissue (0.1 cm x 1 cm x 1 cm) from each of the four inoculation points was excised from the inoculated nectarines for examination by light microscopy. To this end, each sample was fixed by immersion in a solution of formaldehyde-acetic acid-alcohol (ethanol: acetic acid: formaldehyde: water, 50:10:35:5, v/v/v/v). To facilitate fixative infiltration, the samples were first degassed by maintaining them in a mild vacuum for two hours and then overnight incubation in a formaldehyde-acetic acid-alcohol solution at 4°C. Following the overnight incubation, the samples were first gradually dehydrated in graded ethanol (50, 70, 80, and 100%—once for 60 minutes at each step) and then soaked in a graded series of ethanol:Histoclear II (National Diagnostics, Atlanta, GA, USA) (3:1, 1:1, 1:3, 0:1 v/v—once for 120 minutes at each step) to remove the alcohol. The samples were then embedded in paraffin by first immersing each sample in a graded series of molten paraffin: Histoclear II at 60°C (Histo-comp, Casa Alvarez, Madrid, Spain) (1:3, 1:1, 3:1 v/v—once for 60 minutes at each step) and then in molten paraffin for at least 48 hours. Five sections (15-μm thick) of each sample were prepared using a microtome (Microtome pfm medical ag, Köln, Germany), mounted on glass slides, and then stained with Safranin O-Fast-green stain using a previously described protocol [31] or Calcofluor White fluorescent stain (Sigma-Aldrich Co., St. Louis, MO, USA) according to the manufacturer's instructions. Images of each microsection were examined after their capture by a Leica DFC550 camera, which was attached to a Leica DMRE microscope (Leica Microsystems, Wetzlar, Germany).

### Transmission electron microscopic (TEM) analysis of nectarines with a visible *M*. *fructicola* infection

At the end of the incubation, four samples (2 cm x 2 mm) of the dermal and ground tissues from infected and uninfected nectarines were collected using a sterilized disposable scalpel for examination by TEM. To this end, each samples was first fixed by immersion in modified Karnovsky´s fixative solution which contained 2.5% (v/v) glutaraldehyde and 4% (v/v) paraformaldehyde in sodium phosphate buffer (pH 7.0) at 4°C for six hours, and then washed four times for ten minutes in Karnovsky´s fixative solution. Each sample was postfixed in a 1% (w/v) aqueous osmium tetroxide solution at 20°C for two hours, and then washed three times in SDW. Each sample was gradually dehydrated in graded acetone (30%, 50%, 70%, 80%, and 95%—once for 15 minutes at each step), twice in 100% acetone for 15 minutes, embedded in Spurr resin, and polymerized at 65°C for 48 hours according to a previously described protocol [26]. Ultrathin sections (15-μm thick) of each sample was prepared using an ultramicrotome with a diamond knife (Ultracut Reichert, Vienna, Austria) and then mounted on copper grids. The grids were counterstained with 2% (w/v) uranyl acetate for 20 minutes and with Reynolds´ lead citrate for four minutes before being examined using a TEM (JEM-1010, LEOL Ltd., Tokyo) which was operated at 80 kV. At least four embedded blocks were prepared for each incubation time, and more than ten ultrathin sections per block were examined by TEM.

## Results

### Conidial germination and hyphal growth on the nectarine's epidermis

**Fig 1:**
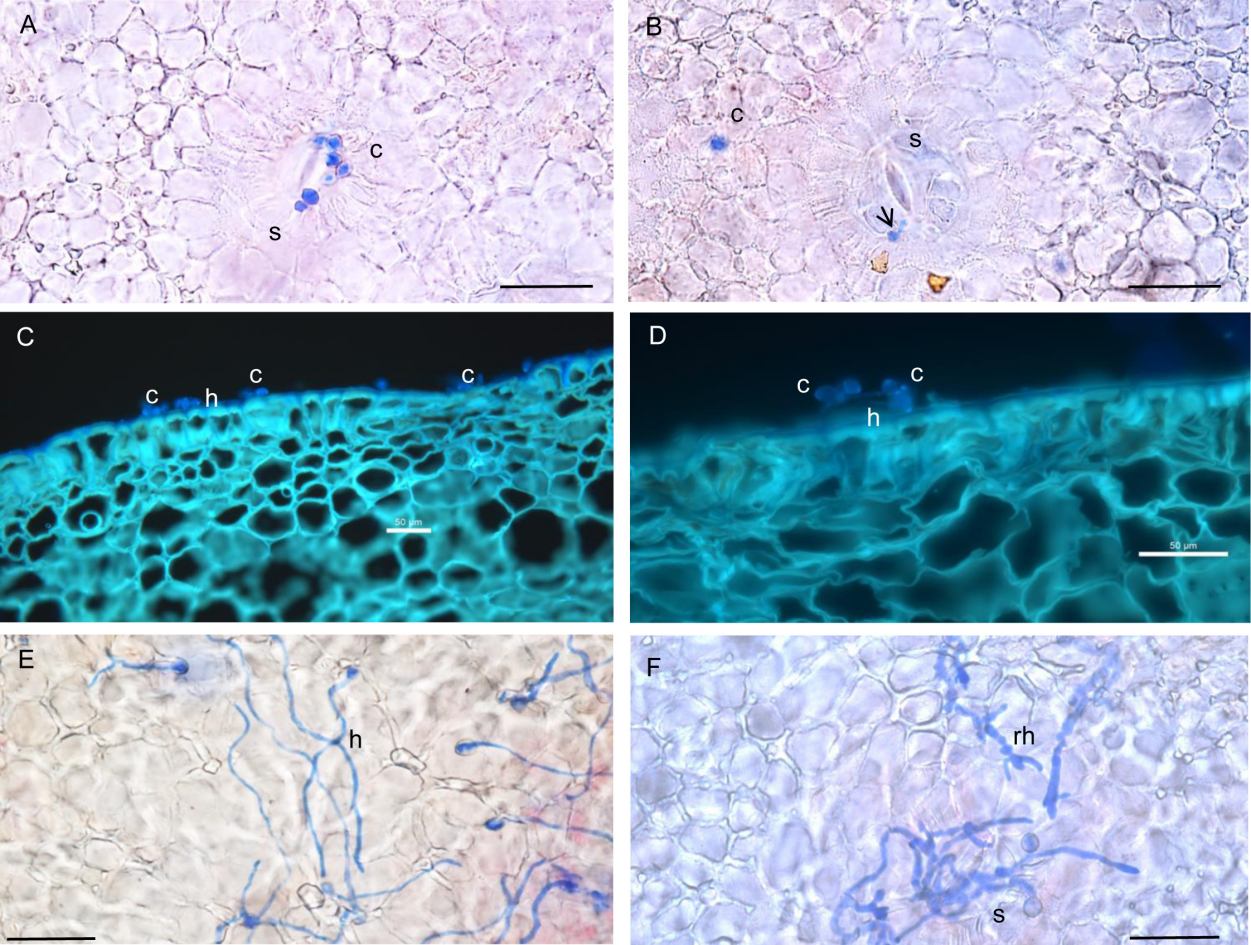
Germination on and penetration of the surface of mature nectarines by *M*. *fructicola* conidia and hyphae. The nectarine’s tissues and fungal structures were stained with lactophenol blue (A, B, E and F) or Calcofluor White fluorescent stain (C and D). A-B: Clusters of non-germinated (blue) and germinated (arrow) *M*. *fructicola* conidia (c) around the guard cells of stomata (s) on the surface of mature fruit after a 4-hour incubation at 25°C. C and D: Conidia and long germ tubes (h) on the surface of mature fruit after a 16-hour incubation at 25°C. Scale bar = 50 μm. E: Long germ tubes on the surface of mature fruit after a 24-hour incubation at 25°C. F: thick ramified hyphae (rh) on the surface of mature fruit after a 24-hour incubation at 4°C.

Fig 1 contains representative light microscopic images of *M*. *fructicola* germination and hyphal development on the nectarine's epidermis after a 4-hour and 24-hour incubation at 25°C or 4°C. Clusters of *M*. *fructicola* conidia were observed around the guard cells of the stomata and some of these conidia had germinated four hours after inoculation when the nectarines were incubated at 25°C (Fig 1A and 1B). The germ tubes grew progressively into long hyphae on the surface between a 4-hour, a 16-hour (Fig 1C and 1D), and a 24-hour (Fig 1E) incubation at 25°C (Table 1), and the hyphae penetrated the surface mostly through stomata (Fig 1B and 1F). A lower number of conidia were observed in areas without stomata (Fig 1A and 1D). When the inoculated nectarines were incubated at 4°C, thick ramified hyphae were observed on the surface after a 24-hour incubation (Fig 1F). Appressoria were not seen on the surface or in the tissues after a 4-hour and 24-hour incubation at 25°C or 4°C.

**Table 1:** Timeline of tissue changes in nectarines with a visible and a latent *M*. *fructicola* infection.

		Initial observations of the infection process 24 hours after inoculation at 25°C (t = 0)	Times of appearance of signs of visible infection (hours after t = 0, at 25°C)			Times of appearance of signs of latent infection (hours after t = 0, at 4°C)			
			24	48	72	72	144	216	288
Visible signs of disease	*Epidermal discoloration*	-	+	+	+	-	-	-	-
	*Brown spots and brown rot*	-	+	+	+	-	-	-	-
	*Sporulation*	-	-	-	+	-	-	-	-
	*Diameter of macroscopic lesion (cm)*	-	1	2.5	5	-	-	-	-
Fungal infection structures and their location	*Conidial germination on fruit surface*	+	+	+	+	-	-	-	-
	*Germ tube growth*	+	+	+	+	-	-	-	-
	*Thin hyphae in the substomatal cavity*	+	+	+	+	+	+	+	+
	*Thin hyphae in the epidermis*	-	+	+	+	-	-	+	+
	*Thin hyphae in the mesocarp*	-	-	+	+	-	-	-	-
	*Thick hyphae in the epidermis and mesocarp*	-	-	-	+	-	-	-	-
	*Sporulation*	-	-	-	+	-	-	-	-
Light microscopic analysis	*Partial cuticular degradation*	-	+	+	+	-	-	-	-
	*Cell degradation in the substomatal cavity*	+	+	+	+	-	+	+	+
	*Collapse and disruption of epidermal cells*	-	+	+	+	-	-	+	+
	*Formation of lysogenic cavities at subepidermal level*	-	-	+	+	-	-	-	-
	*Collapse and disruption of mesocarpic cells*	-	-	+	+	-	-	-	-
	*Formation of lysogenic cavities at mesocarpic level*	-	-	+	+	-	-	-	-
	*Cellular degradation and cuticular breakdown*	-	-	-	+	-	-	-	-
Transmission electron microscopic analysis	*Germination and adherence of conidia to fruit surface*	+		+					
	*Thin intramural hyphae in epidermal cells; cell wall degradation*	+		+					
	*Thick intercellular and intracellular hyhae in mesocarpic cells; cell wall degradation and cell death*	-		+					

Table 1 summarizes the timeline of tissue changes in nectarines with a latent or a visible *M*. *fructicola* infection according to the results of the light microscopic and TEM analyses. For nectarines with visible brown rot, the signs of brown rot appeared within 48 hours on the surface of infected nectarines as brown spots on the surface at the inoculation sites and then became smooth grey spots after 72 hours. Thereafter, the disease developed over the surface and in the parenchyma. For nectarines with a latent infection, no signs of disease were seen on the nectarine's surface over the 288-hour incubation period at 4°C.

### Light microscopic analysis of tissues from nectarines with a visible *M*. *fructicola* infection

**Fig 2:**
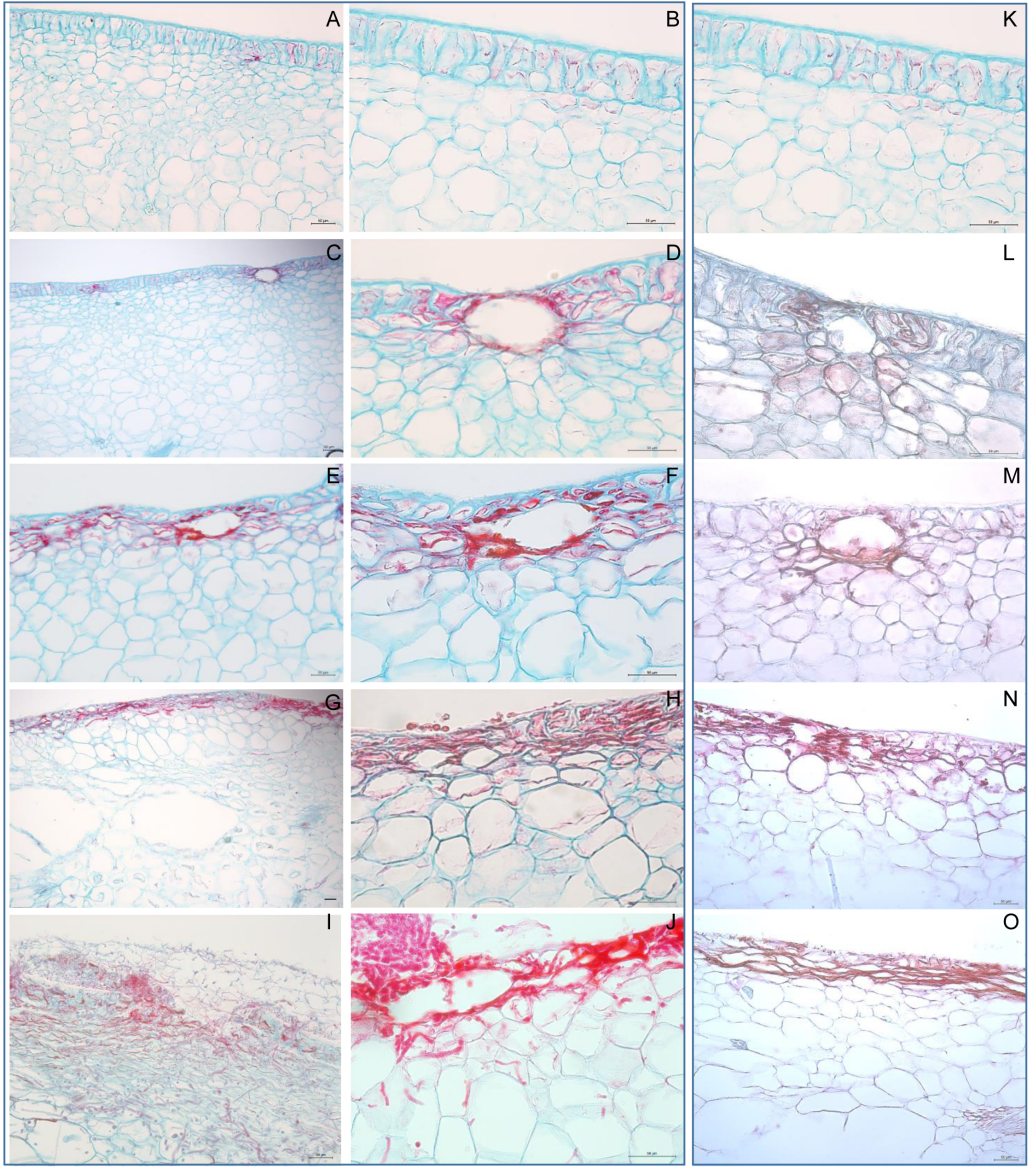
Light microscopic analysis of tissues from nectarines with a visible or latent *M*. *fructicola* infection. The nectarine's tissues were stained with Safranin O-fast Green stain and the unaffected walls of the nectarine's cells are blue and the affected nectarine’s cells, and the hyphae and conidia of *M*. *fructicola* are red: A, B and K: representative images of epidermal, subepidermal and mesocarp cells from uninoculated nectarines; C and D: signs of *M*. *fructicola* infection in areas which surround the stomata and in stomata-free areas of nectarines with a visible infection after a 24-hour incubation at 25°C. E and F: invasion and collapse of the uppermost layer of epidermal cells of nectarines with a visible infection after a 48-hour inoculation at 25°C. G and H: Collapse of epidermal cells, apparition of lysogenic cavities in the mesocarp, and extensive colonization of the deep subdermal tissues by *M*. *fructicola* of nectarines with a visible infection after a 72-hour incubation at 25°C. I and J: Colonization of the epidermis and mesocarp by *M*. *fructicola* with thin and thick hyphae, collapse and disruption of the nectarine's epidermal and mesocarpic cells, lysogenic cavities in the subepidermis, degradation of the cuticle and epidermis, and *M*. *fructicola* sporulation after a 96-hour incubation at 25°C. L: Colonization of the stomata of nectarines with a latent *M*. *fructicola* infection after a 72-hour incubation at 4°C. M: Increased colonization of the stomata of nectarines with a latent *M*. *fructicola* infection after a 144-hour incubation at 4°C. N: Colonization of the subdermal tissues by *M*. *fructicola* with the collapse of the epidermal cells was observed after 216-hour incubation at 4°C. O: Collapse of the epidermal cells and colonization of the subepidermis by *M*. *fructicola* of nectarines with a latent *M*. *fructicola* infection after a 288-hour incubation at 4°C. Scale bar = 50 μm.

Fig 2A and 2B display representative light microscopic images of epidermal, subepidermal, and mesocarp cells from uninoculated nectarines and Fig 2C–2J display representative images of diseased tissue from nectarines with visible signs of a *M*. *fructicola* infection. In nectarines with visible infection, signs of the disease usually appeared after 24 hours on the surface, preferentially in areas which surround stomata but they also appeared in stomata-free areas (Fig 2C and 2D; Table 1). The brown rot became evident in the uppermost layers of epidermal cells below the cuticle and these epidermal cells began to collapse after 48 hours (Fig 2E and 2F; Table 1). The number of collapsed epidermal cells increased after 72 hours and this cellular collapse was accompanied by (a) extensive colonization of the deep subdermal tissues by *M*. *fructicola*, and (b) apparition of lysogenic cavities in the mesocarp (Fig 2G and 2H; Table 1). After 96 hours, the diseased tissues of the nectarines with visible brown rot displayed (a) colonization of the epidermis and mesocarp by *M*. *fructicola* with thin and thick hyphae, (b) collapse and disruption of epidermal and mesocarpic cells, (c) lysogenic cavities in the subepidermis and mesocarp, (d) degradation of the cuticle and epidermis, and (e) *M*. *fructicola* sporulation (Fig 2I and 2J; Table 1).

### Light microscopic analysis of tissues from nectarines with a latent *M*. *fructicola* infection

Fig 2K displays representative light microscopic images of epidermal, subepidermal, and mesocarp cells from uninoculated nectarines and Fig 2L–2O display representative images of the histological changes on the surface and parenchyma of nectarines with a latent *M*. *fructicola* infection. Although no visible signs of infection were seen in nectarines with a latent infection after a 288-hour incubation at 4°C, microscopic signs of the infection became apparent over time. *M*. *fructicola* colonized the stomata of inoculated fruit after 72 hours (Fig 2L; Table 1) and the extent of this stomatal colonization had increased after 144 hours (Fig 2M; Table 1). Colonization of the subdermal tissues by *M*. *fructicola* with the collapse of the epidermal cells was observed after 216 hours (Fig 2N; Table 1). After 288 hours, most of the epidermal cells had collapsed and the subepidermis of the inoculated fruit had become colonized by *M*. *fructicola* (Fig 2O; Table 1).

### TEM analysis of tissues from uninfected nectarines and nectarines with a visible *M*. *fructicola* infection

**Fig 3:**
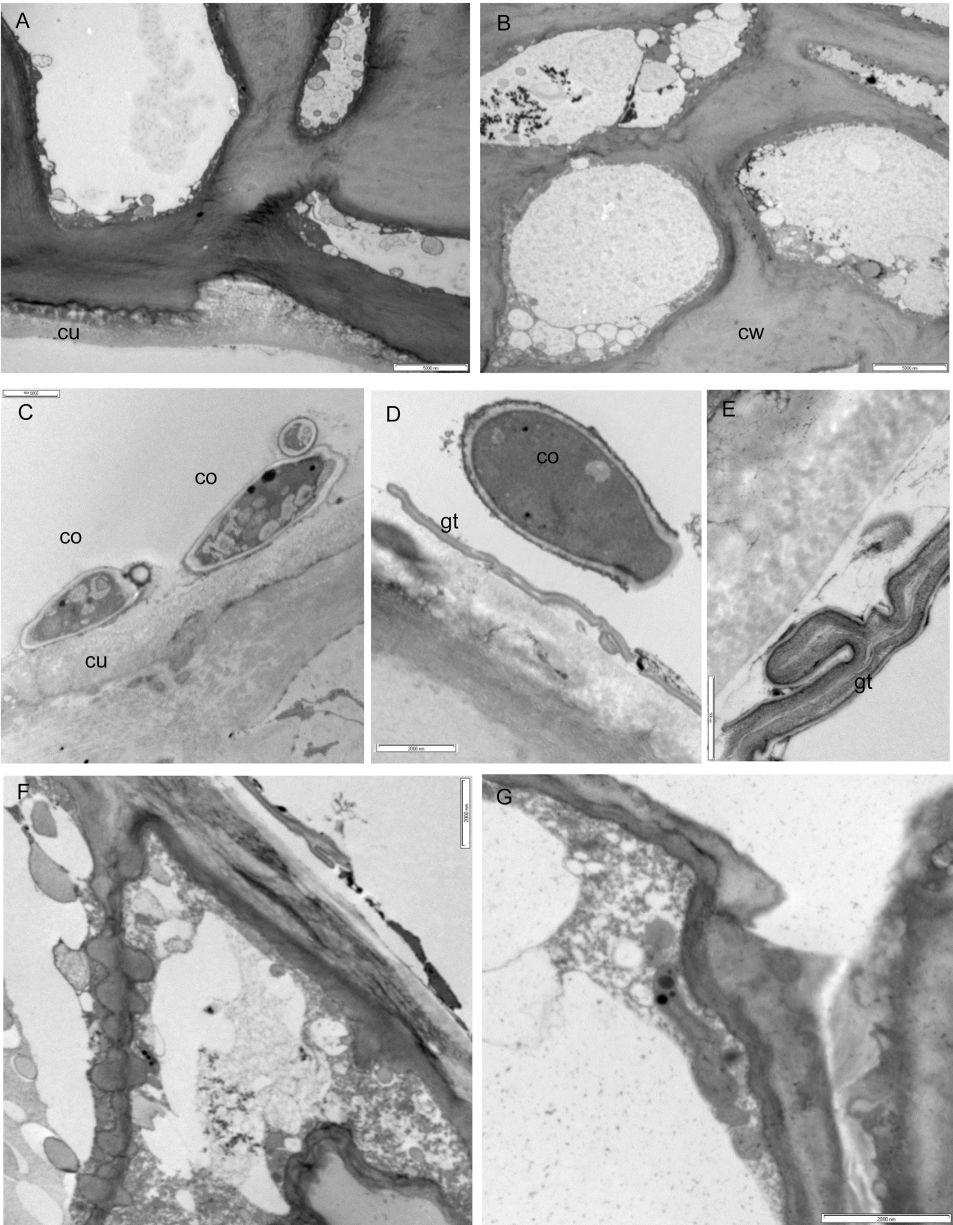
Ultrastructure of tissues from healthy nectarines and nectarines with a visible *M*. *fructicola* infection after a 24-hour incubation at 25°C. A and B: The cuticle (cu), cell wall (cw) and ultrastructure of epidermal and hypodermal cells of healthy nectarines, scale bar = 5000 nm. C-E: Conidial germination (co) and germ tubes (gt) *of M*. *fructicola* on the surface of the cuticle of nectarines with a visible infection, scale bars = 2000 nm (C and D) and 500 nm (E). F and G: Intercellular hyphae (h) *of M*. *fructicola*, scale bar = 2000 nm.

**Fig 4:**
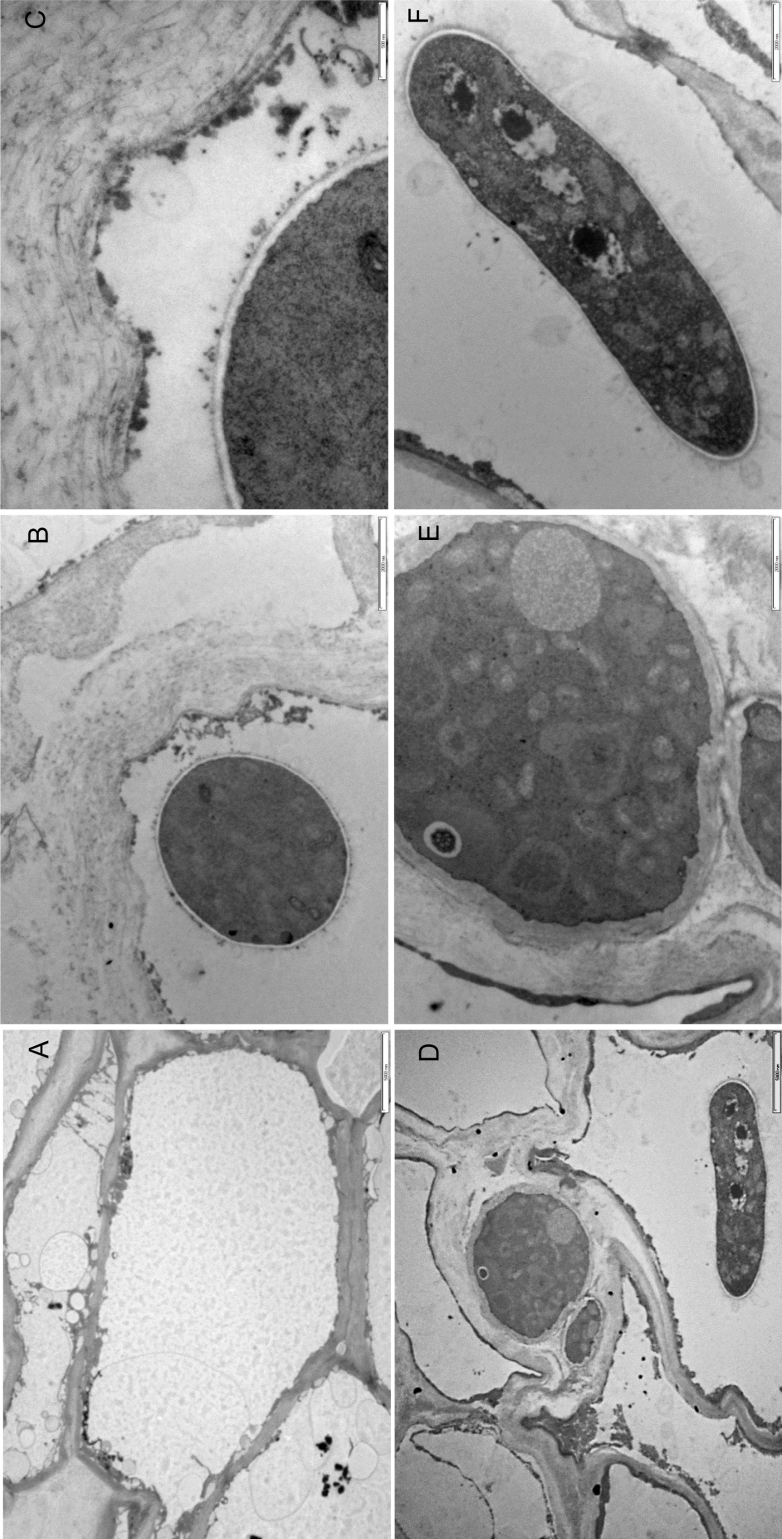
Ultrastructure of tissues from healthy nectarines and nectarines with a visible *M*. *fructicola* infection after a 72-hour incubation at 25°C. A: The parenchyma of an uninfected nectarine, scale bar = 5000 nm. B, C, and F: Intracellular hyphae of *M*. *fructicola* in a mesocarpic cell of a nectarine with a visible infection, scale bars = 2000 nm (B and F) and 500 nm. D: The intracellular and intercellular hyphae of an *M*. *fructicola* in a mesocarpic cell and in the space around a mesocarpic cell of a nectarine with a visible infection, scale bar = 5000 nm. E: The intercellular hypha of *M*. *fructicola* in a mesocarpic cell of a nectarine with a visible infection, scale bar = 2000 nm.

Figs 3 and 4 display representative micrographs of the ultrastructure of healthy nectarines and nectarines with a visible *M*. *fructicola* infection. The cuticle of uninfected fruit had a variable thickness and covered a layer of epidermal cells (Fig 3A). The epidermal and hypodermal cells of healthy nectarines have a thick cell wall of variable thickness and a dense cytoplasm with intact organelles (Fig 3A and 3B). The mesocarpic cells of uninfected nectarines were large with a thin cell wall and the intercellular spaces between the mesocarpic cells were also large. In these cells, the cytoplasm was almost completely occupied by a central vacuole (Fig 4A).

In nectarines with a visible infection, conidia and germ tubes began to adhere to the nectarine's cuticle after 24 hours (Fig 3C–3E). The conidia had a two-layered cell wall, a plasmalemma, and their cytoplasmic matrix was dense with nuclei, mitochondria, and vacuoles (Fig 3C and 3D). Partial degradation and/or dissolution of cuticle and cell wall were observed under germinated *M*. *fructicola* conidia (Fig 3C–3E). Long branching germ tubes were present on the cuticular surface and some of these tubes had penetrated the cuticle presumably through the stomatal cavities into epidermal cells (Fig 3E). Thin infective hypha that grew intercellularly (Fig 3F) or intracellularly (Fig 3G) in epidermal cells were observed after 24 hours. Substantial dissolution of the cell walls and degeneration of cytoplasmic organelles were evident in infected epidermal and hypodermal cells (Fig 3F and 3G). Hyphal invasion, cytoplasmic necrosis, and degeneration of cytoplasmic organelles in mesocarpic cells were observed after 72 hours (Fig 4B–4F). The mesocarpic cells were colonized by large intercellular (Fig 4B–4F) and intracellular hyphae (Fig 4B–4D and 4F). Intracellular hyphae, which were close to the cell wall, contain numerous organelles in their cytoplasm, have a double cell wall layer, and are surrounded by vesicles (Fig 4B). The matrix of the mesocarpic cell walls, which were close to intracellular hyphae, appeared fibrillary and disrupted, and groups of vesicles were present near the mesocarpic cell walls (Fig 4B and 4C). The intercellular hyphae, which had invaded mesocarpic cells, were ultrastructurally different from the intracellular hyphae: their cytoplasm contained no vesicles and their cell walls were thick (Fig 4D and 4E).

## Discussion

The aim of this investigation was to analyze the microanatomy of nectarines with a latent and a visible *M*. *fructicola* infection using light microscopy and TEM. Tissues of mature nectarines with latent *M*. *fructicola* infections are characterized by the presence of intercellular hyphae at the subcuticular level. Although these intercellular hyphae do not penetrate more than the first two subcuticular cell-layers of nectarines with latent infections, they do not remain totally dormant but slowly colonize the tissue. At the same time, the tissues of mature nectarines with visible *M*. *fructicola* infections are characterized by extensive colonization of the deep subdermal tissues by the hyphae of *M*. *fructicola*. The tissues and the cells were colonized inter- and intracellularly and this colonization was accompanied by increasing degradation of the cell walls, a typical colonization of necrotrophic fungi.

Germinated conidia with long germ tubes have been described in mature nectarines with brown rot due to *M*. *fructicola* and this germination is associated with a low stomatal density, reduced hydrophobicity of the cuticle, numerous cuticular cracks and fissures, and the accumulation of volatile compounds [9]. In our study, we observed long germ tubes of *M*. *fructicola* around the guard cells of stomata on the surface of mature fruit mainly when samples of the epidermis from inoculated nectarines were incubated at 25°C. For *Monilinia* spp., conidial germination, sporulation, growth, and propagule formation on fruit surfaces are dependent on T, RH, and water availability [11,32–36]. We found germination of *M*. *fructicola* conidia and hyphal formation on the nectarine surface following a 4-hour incubation at 25°C and a 24-hour incubation at 4°C. We also found that this germination and hyphal formation were more abundant in areas around the stomata than in areas without stomata. Our results on *M*. *fructicola* germination on the fruit surface are similar to those of other investigators who reported that *Monilinia* spp. can penetrate stone fruit through the stomata, the cuticle of the intact surface, the base of the surface hairs and trichomes, and surface wounds [6,37–39].

We did not detect appressoria on the surface or inside epidermal cells of mature nectarines with either a visible or latent *M*. *fructicola* infection. Other investigators have reported the presence of appressoria on immature fruit and flowers. For example, Lee and Bostock [9] described appressorial formation by *M*. *fructicola* on immature nectarine surfaces. *M*. *fructicola* forms non-melanized appressoria for penetrating *Prunus* spp. petals and immature fruit, and appresoria has been proposed as resting structures of latent infections in immature fruit [40]. Other factors, such as unidentified volatile and nonvolatile compounds in the cuticle and/or cell wall, have been implicated for stimulating of appressorial formation by fungal pathogens [41,42].

Although visible evidence of brown rot was not seen on the surface of nectarine with a latent infection, we detected fungal hyphae in the epidermal cells after 144 hours of incubation at 4°C when the tissues of these nectarines were microscopically examined. Rungjindamai et al. [43] suggested that latent infections follow a typical pattern of subcuticular infection of immature fruit followed by rapid cessation of growth of the pathogen. In this investigation, we found slow and continuous growth of the pathogen in nectarine subcuticular tissues with a latent infection under conditions of cold storage. Although no macroscopic brown rot symptoms are observed, *Monilinia* hyphae were visible at subcuticular level and the latent infection could be considered a quiescent infection under optical microscopy conditions according to the definition of Ahimera et al. [14]. It has been reported that activation of a latent infection can be facilitated by large gene families of cell wall-degrading enzymes [44]. Additionally other pathogenic fungi use tissue acidification to activate a latent infection and stimulate the transition of a latent infection to a visible infection [44]. It has also been reported that *M*. *fructicola* can acidify the tissues of the host and hence up-regulate the expression of polygalacturonase genes, which is controlled by acidic pH as the infected fruit matures [45]. We found cell wall degradation in the epidermal and mesocarpic cells in the nectarines with a visible *M*. *fructicola* infection. We also found (a) partial degradation or dissolution of cuticle and cell wall areas under germinated conidia, (b) collapse of epidermis cells, and (c) formation of lysogenic cavities on the nectarine mesocarp 72 hours after inoculation from visible *M*. *fructicola* infections. It has been reported that *M*. *fructicola* produces cell degrading enzymes, such as cutinase [46], and that this cutinase is important for *M*. *fructicola* virulence during fungal growth and the development of brown rot lesions in nectarines [47]. It has also been reported that the redox state of nectarines can influence appressorial formation and expression of the *M*. *fructicola* cutinase *Mfcut1* and polygalacturonase *Mfpg1* genes [40]. Interestingly, Wade and Cruickshank [37] reported a thick mechanical barrier around the infection point, suberized cell walls of the surrounding living cells, and intracellular accumulation of phenolic compounds in apricots with a latent *M*. *fructicola* infection.

We detected that *M*. *fructicola* only colonizes the intercellular spaces at subcuticular level in nectarines with a latent infection, whereas hyphae colonize inter- and intracellularly in nectarines with a visible infection. These findings are similar to those which have been reported for corn: the hyphae of *F*. *moniliforme* and *F*. *verticillioides* colonize the intercellular spaces in latent infections, whereas their hyphae colonize intracellularly in visible infections [48–50]. Other pathogenic fungi, such as *Phialophora gregata* in soybean [21], or *Colletotrichum acutatum* in their solanaceous hosts [51], have been reported to colonize and survive intercellularly.

A latent brown rot infection is a well-known but poorly understood phenomenon. In this investigation, we found that (a) intercellular hyphal colonization was restricted to the epidermal and two subdermal cell layers in nectarines with a latent *M*. *fructicola* infection, and (b) no macroscopic evidence of disease was detected in latently infected nectarines even after 216 hours of incubation at 4°C. We also found that intra- and intercellular hyphae colonized all mesocarp cells in nectarines with visible brown rot. Finally, we also found that *M*. *fructicola* is active during latent infections because slow and progressive colonization of nectarine cells by the fungus occurs.

## Supporting Information

S1 Fig Germination on and penetration of the surface of mature nectarines by *M*. *fructicola* after 4 hours of incubation at 25°C. (TIF)

S2 Fig Germination on and penetration of the surface of mature nectarines by *M*. *fructicola* after 24 hours of incubation at 25°C. (TIF)

S3 Fig Germination on and penetration of the surface of mature nectarines by *M*. *fructicola* after 24 hours of incubation at 4°C. (TIF)

S4 Fig Light microscopic analysis of tissues from nectarines without *M*. *fructicola* infection. (TIF)

S5 Fig Light microscopic analysis of tissues from nectarines with a visible *M*. *fructicola* infection after 24 hours of incubation at 25°C. (TIF)

S6 Fig Light microscopic analysis of tissues from nectarines with a visible *M*. *fructicola* infection after 48 hours of incubation at 25°C. (TIF)

S7 Fig Light microscopic analysis of tissues from nectarines with a visible *M*. *fructicola* infection after 72 hours of incubation at 25°C. (TIF)

S8 Fig Light microscopic analysis of tissues from nectarines with a visible *M*. *fructicola* infection after 96 hours of incubation at 25°C. (TIF)

S9 Fig Light microscopic analysis of tissues from nectarines with a latent *M*. *fructicola* infection after 144 hours of incubation at 4°C. (PDF)

S10 Fig Light microscopic analysis of tissues from nectarines with a latent *M*. *fructicola* infection after 256 hours of incubation at 4°C. (PDF)

S11 Fig Light microscopic analysis of tissues from nectarines with a latent *M*. *fructicola* infection after 288 hours of incubation at 4°C. (PDF)

S12 Fig Ultrastructure of tissues from healthy nectarines. (TIF)

S13 Fig Ultrastructure of tissues from nectarines with a visible *M*. *fructicola* infection after a 72-hour incubation at 25°C. (TIF)
