# Supplementary figures and images for: Microscopic Analyses of Latent and Visible Monilinia fructicola Infections in Nectarines
Source: PLoS One. 2016 Aug 5;11(8):e0160675. doi: 10.1371/journal.pone.0160675 (PMC4975509; doi:10.1371/journal.pone.0160675)

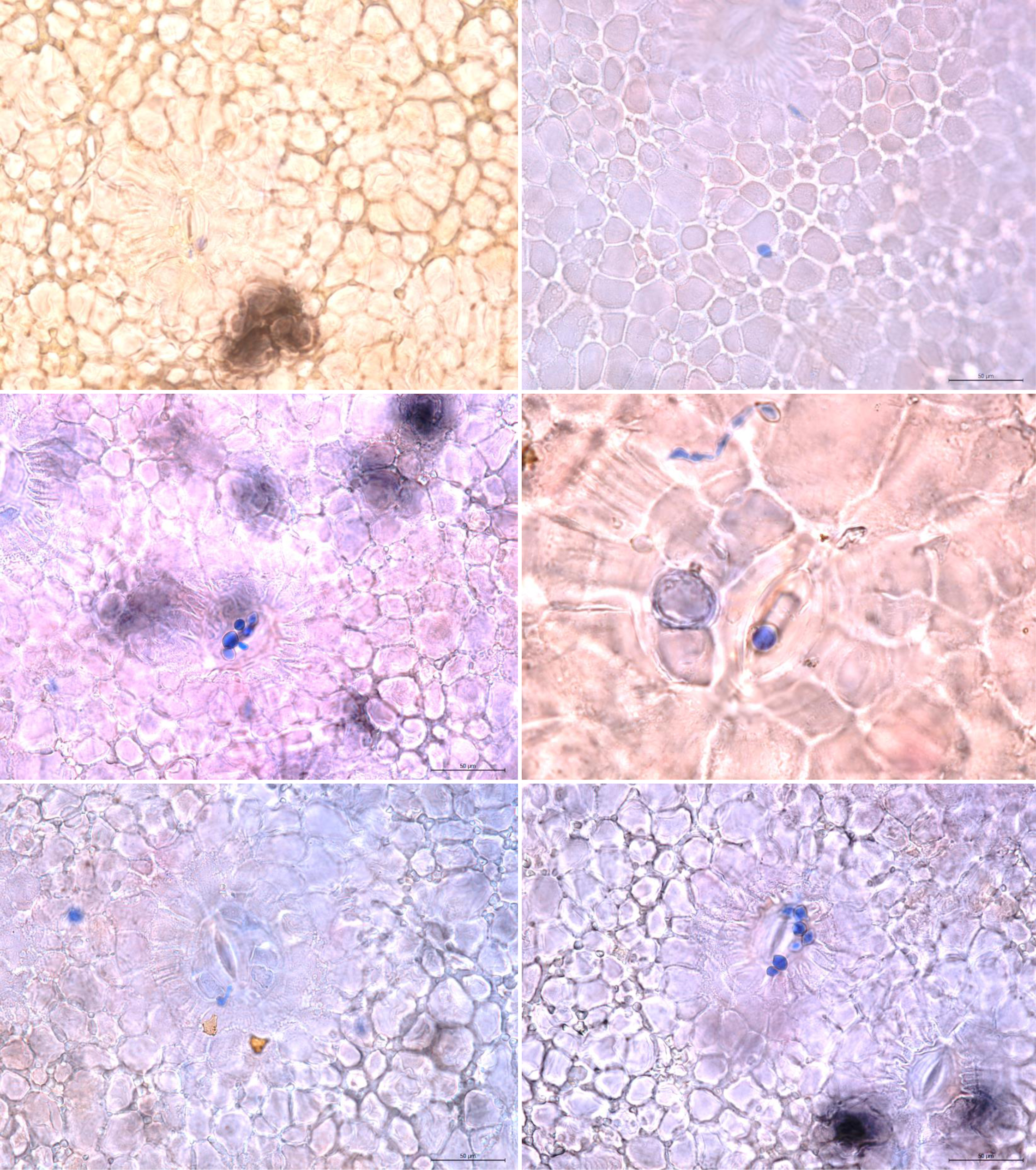

Supplement: S1 Fig — (TIF) [file pone.0160675.s001.tif]

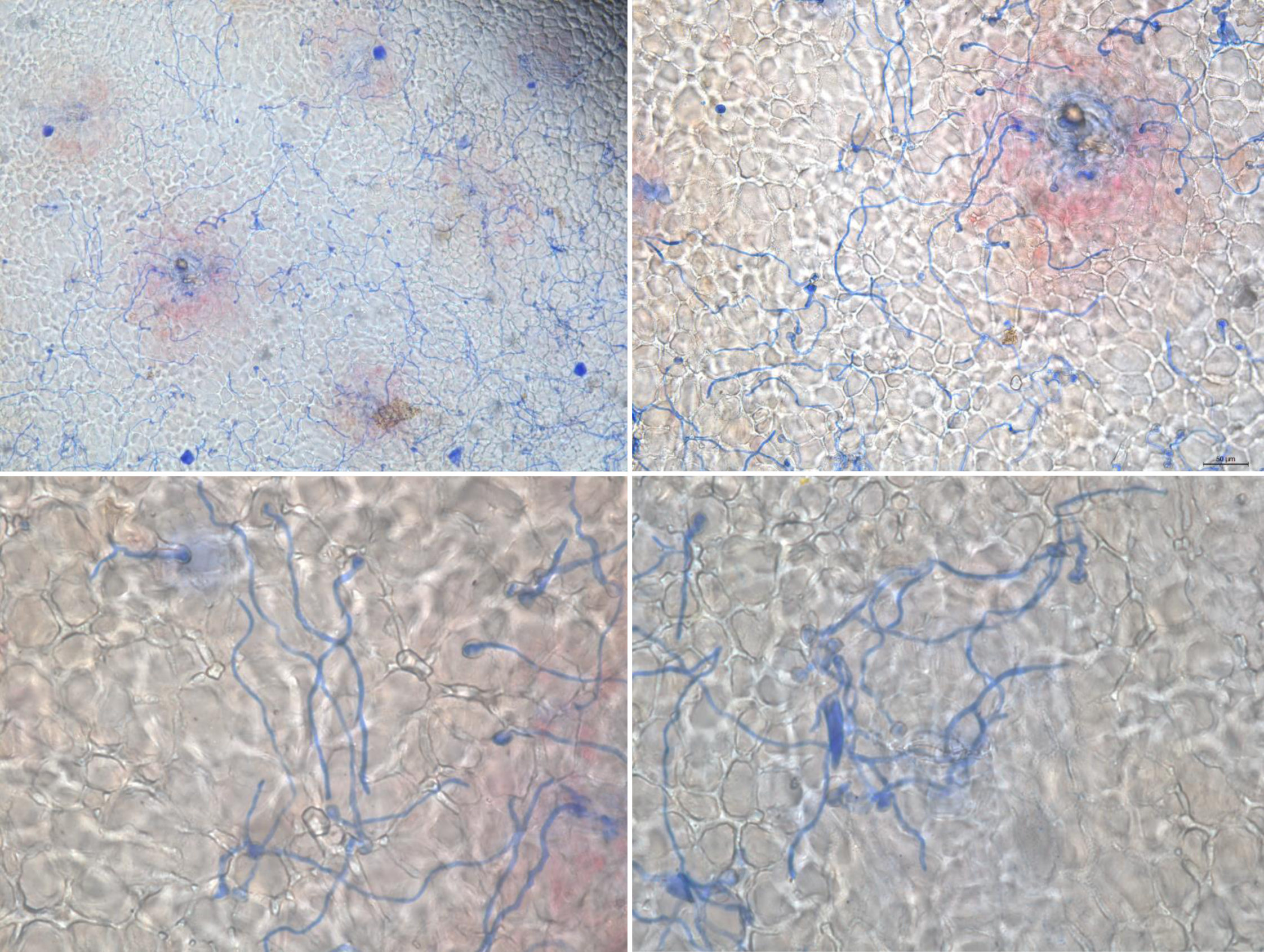

Supplement: S2 Fig — (TIF) [file pone.0160675.s002.tif]

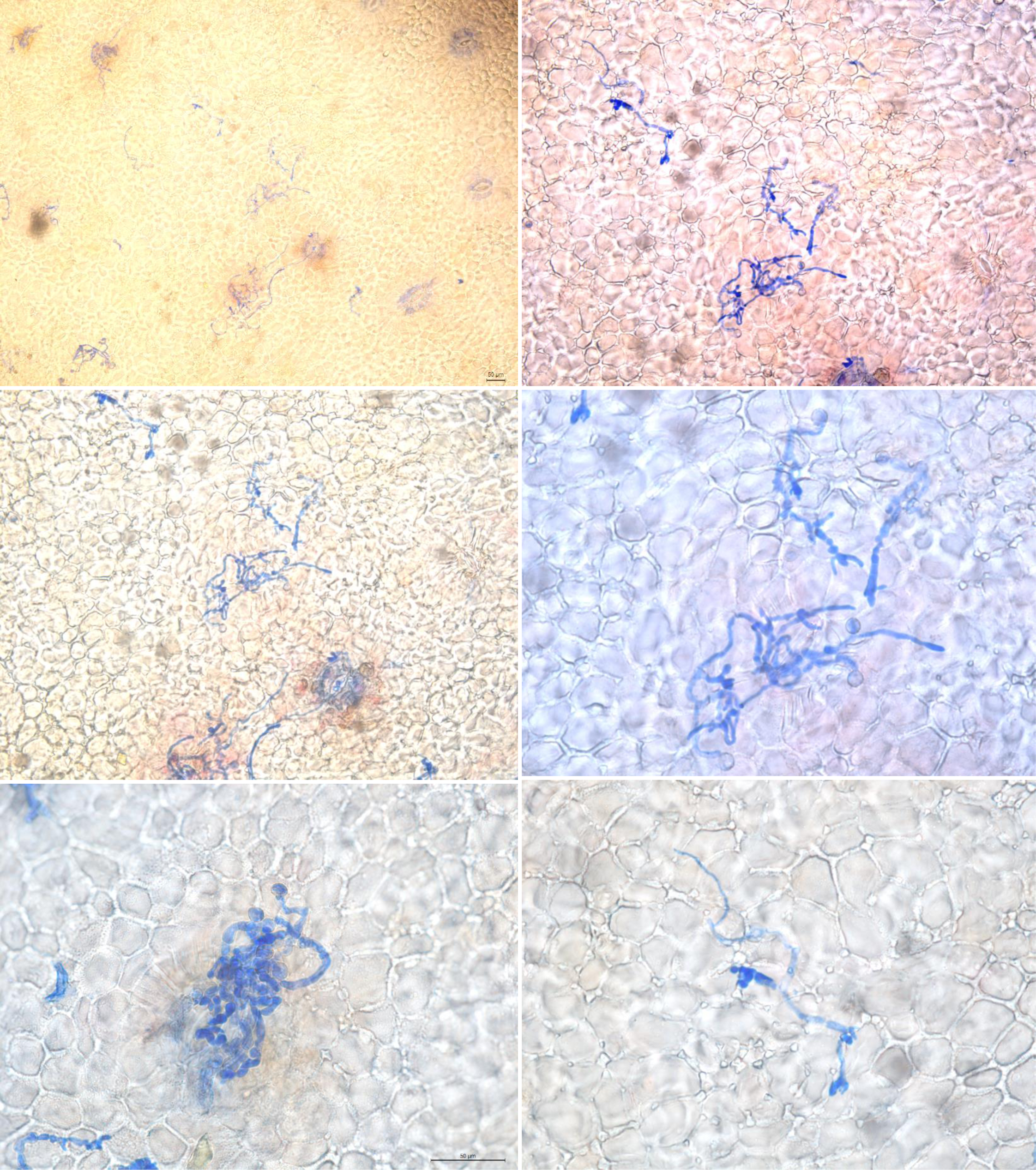

Supplement: S3 Fig — (TIF) [file pone.0160675.s003.tif]

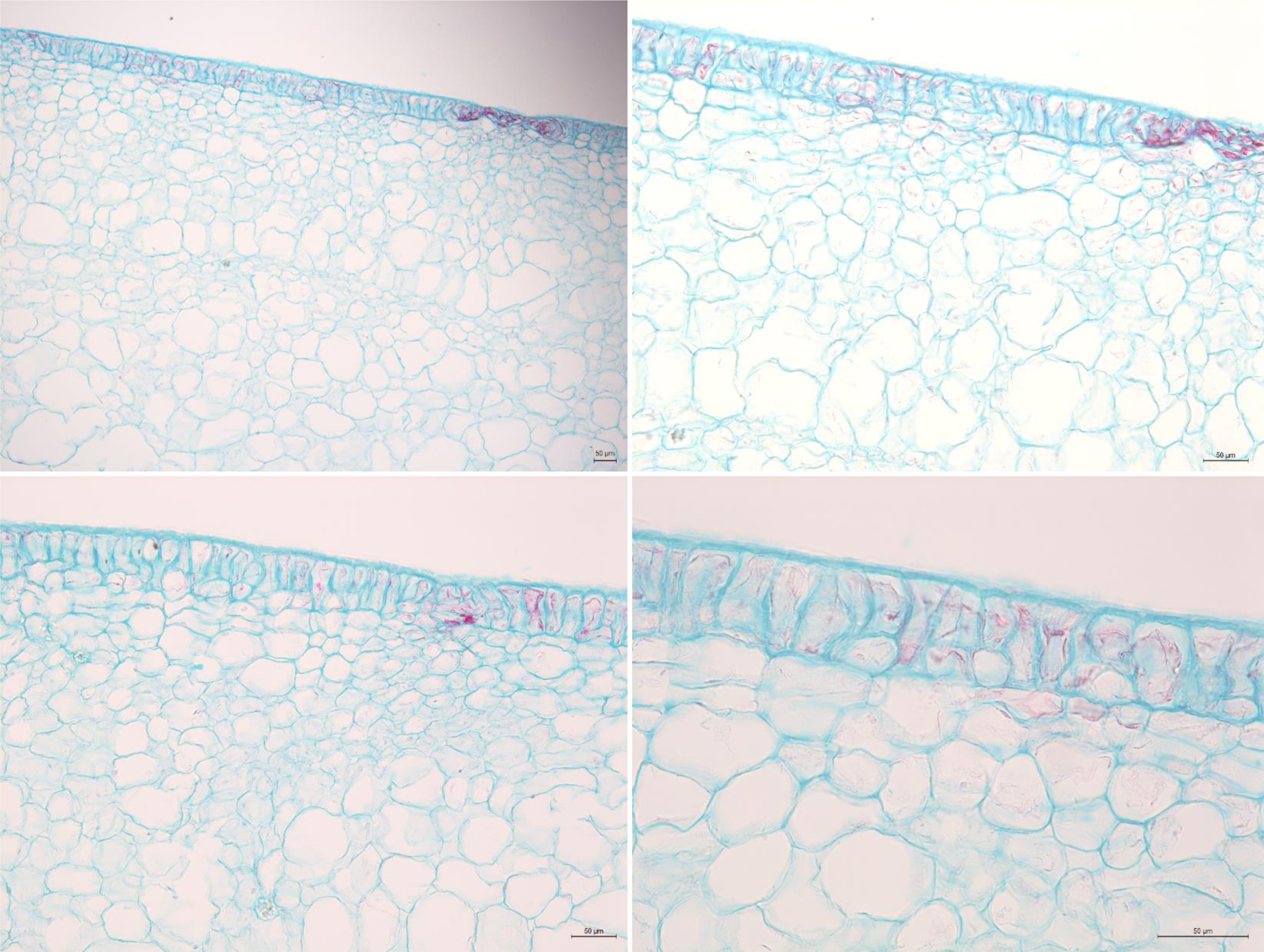

Supplement: S4 Fig — (TIF) [file pone.0160675.s004.tif]

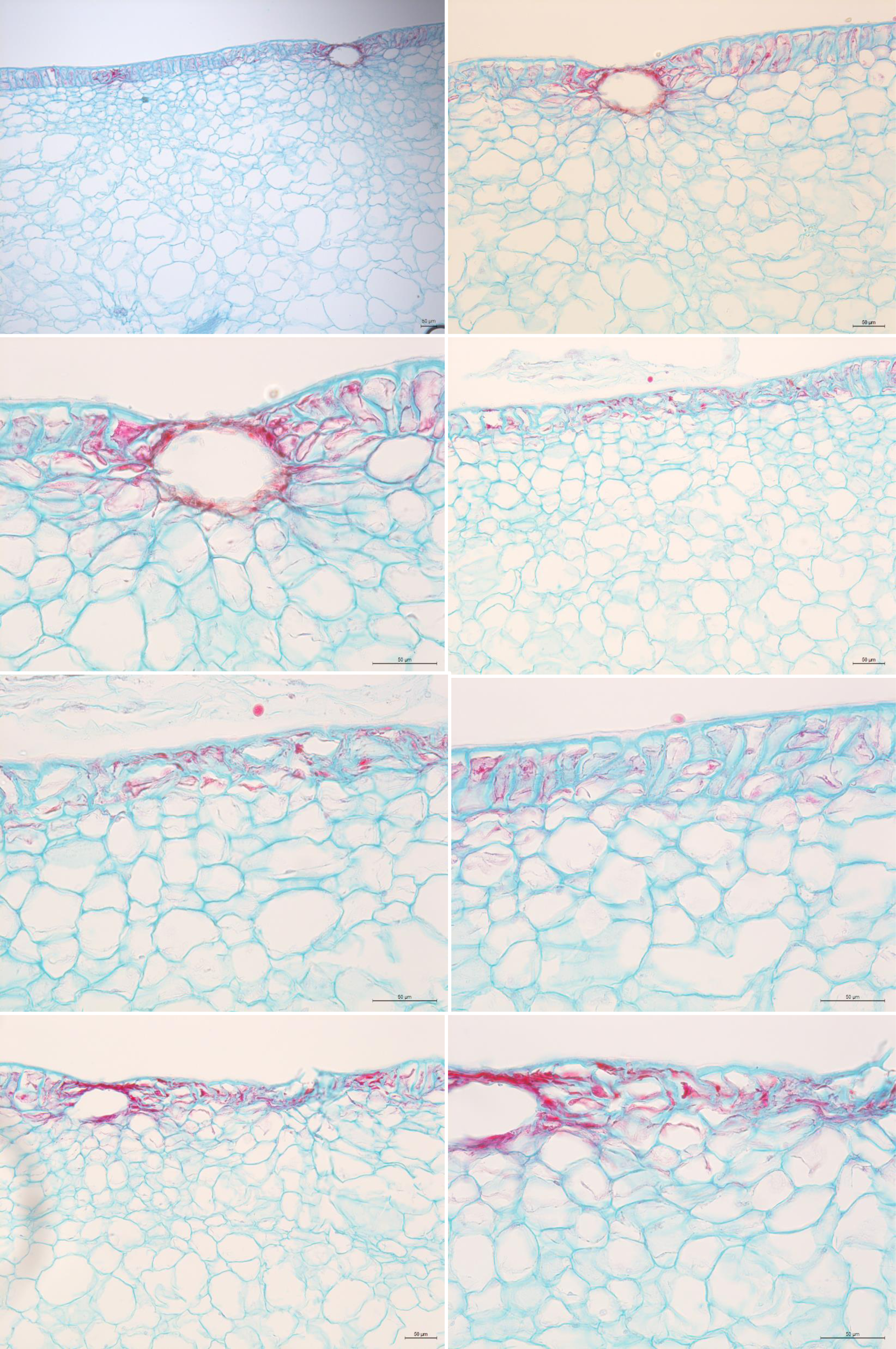

Supplement: S5 Fig — (TIF) [file pone.0160675.s005.tif]

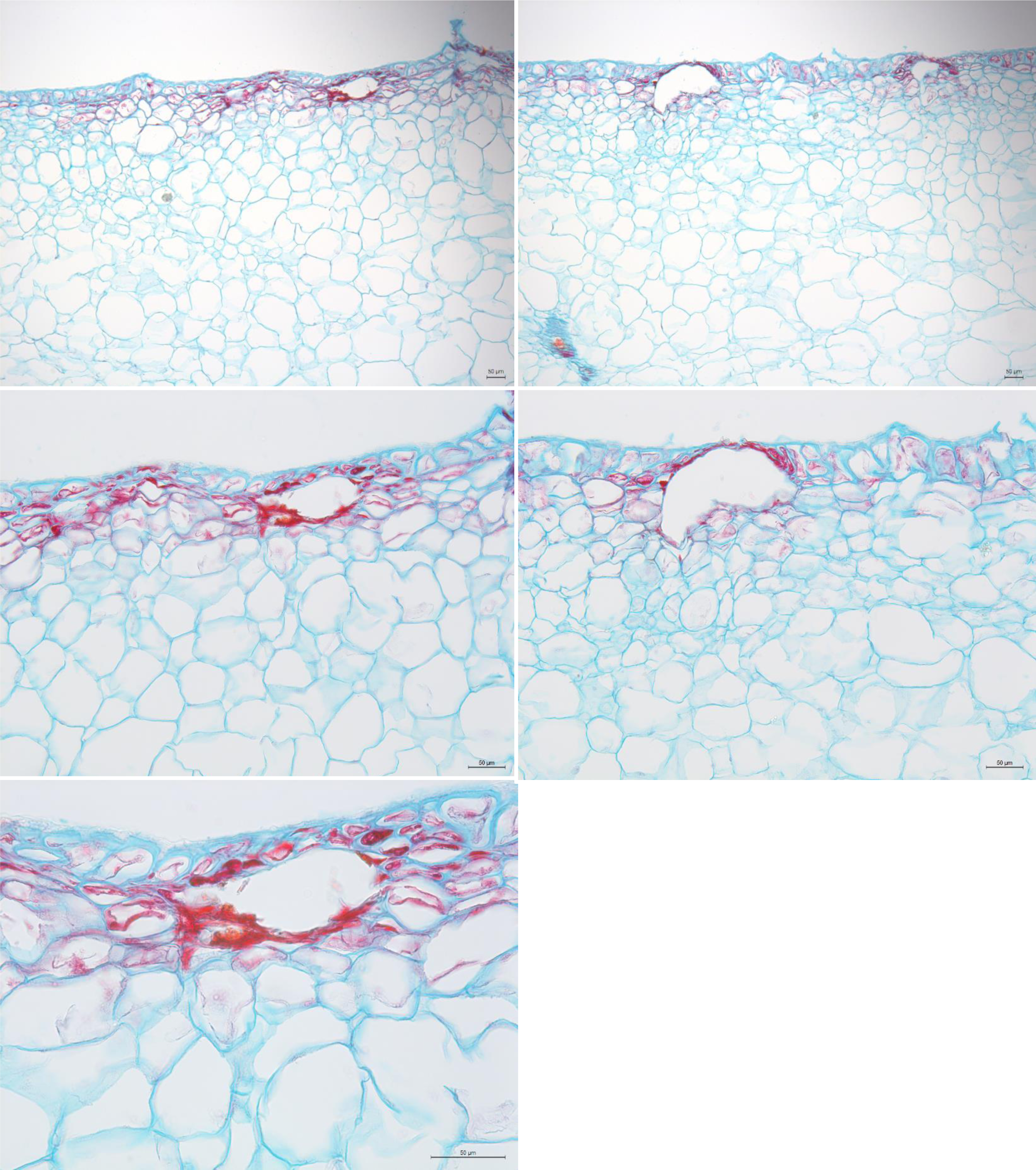

Supplement: S6 Fig — (TIF) [file pone.0160675.s006.tif]

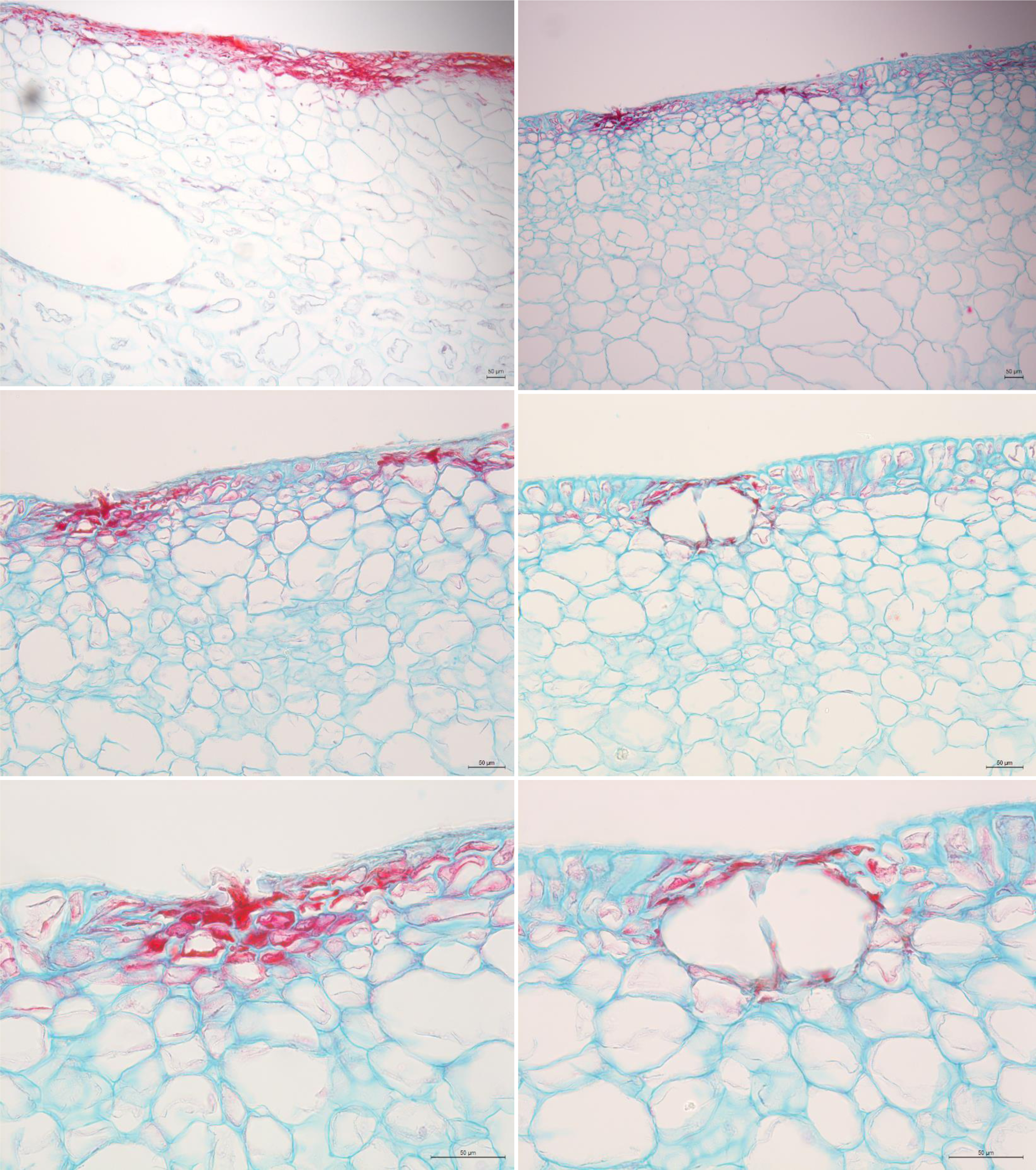

Supplement: S7 Fig — (TIF) [file pone.0160675.s007.tif]

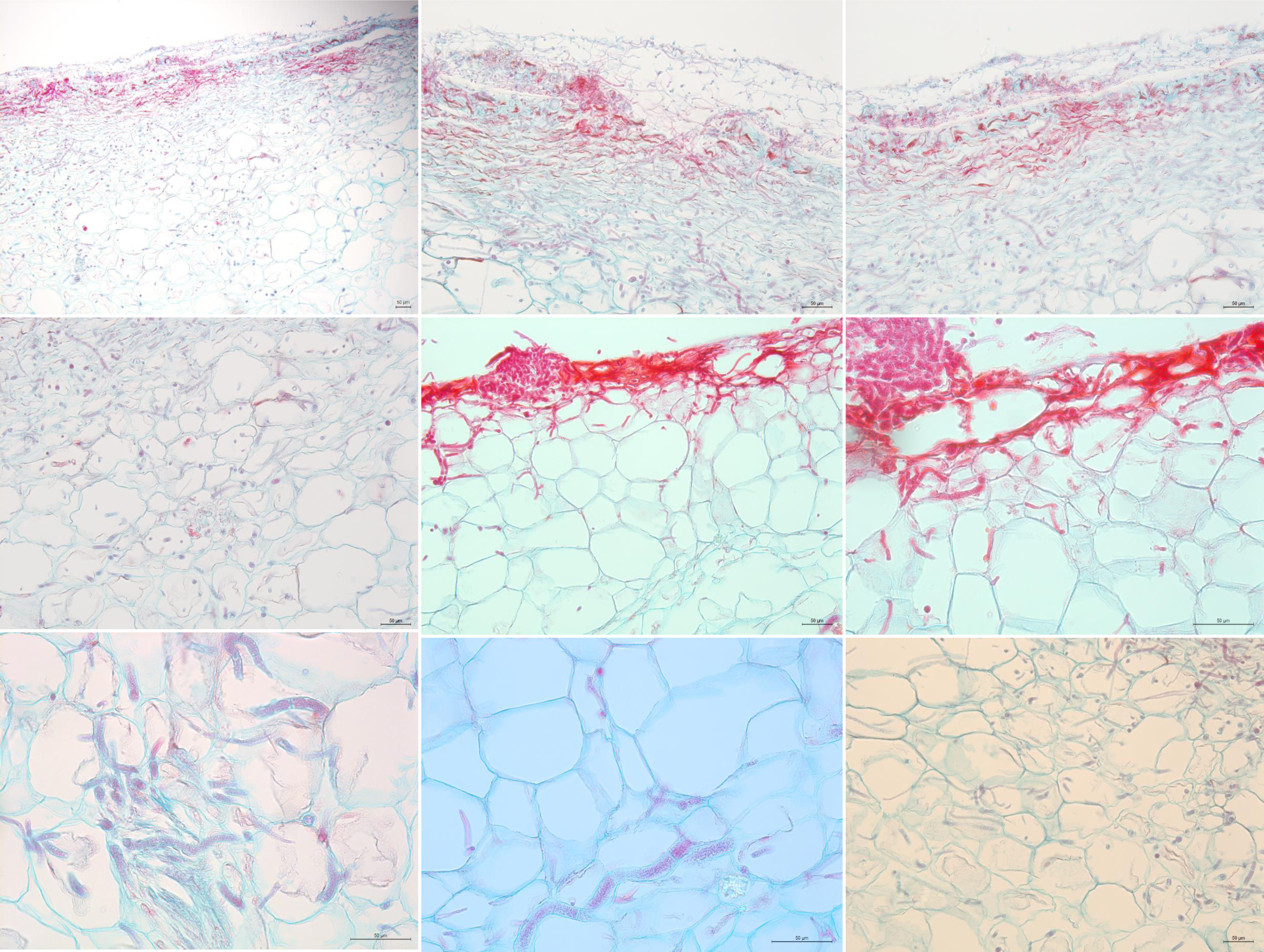

Supplement: S8 Fig — (TIF) [file pone.0160675.s008.tif]

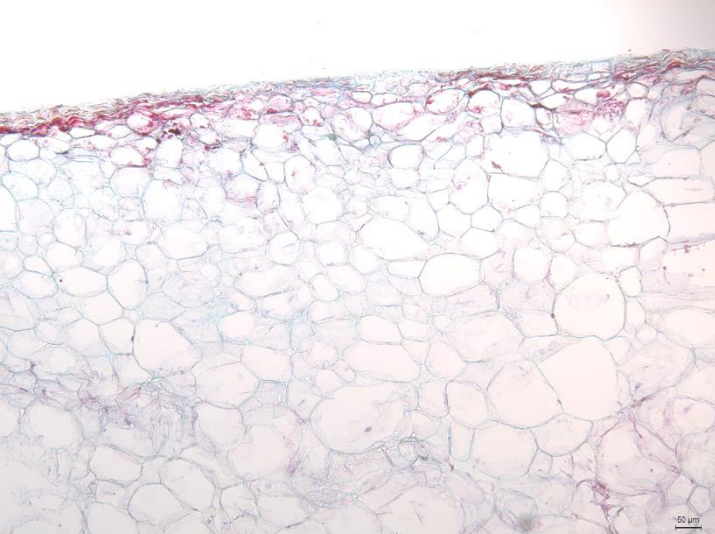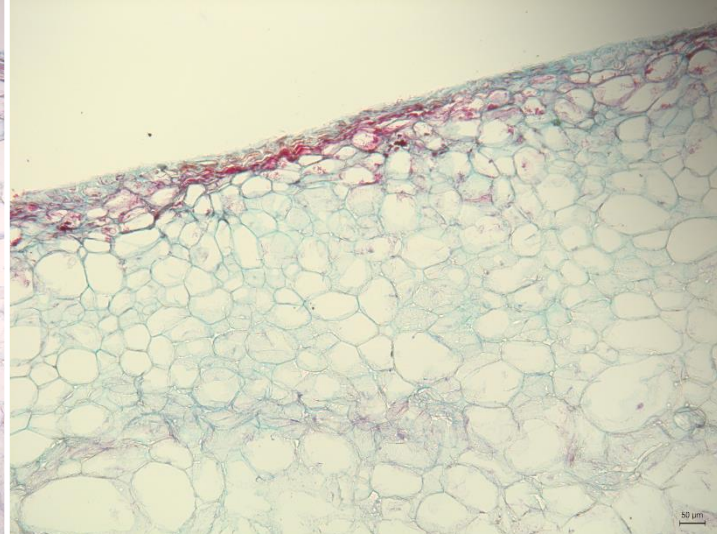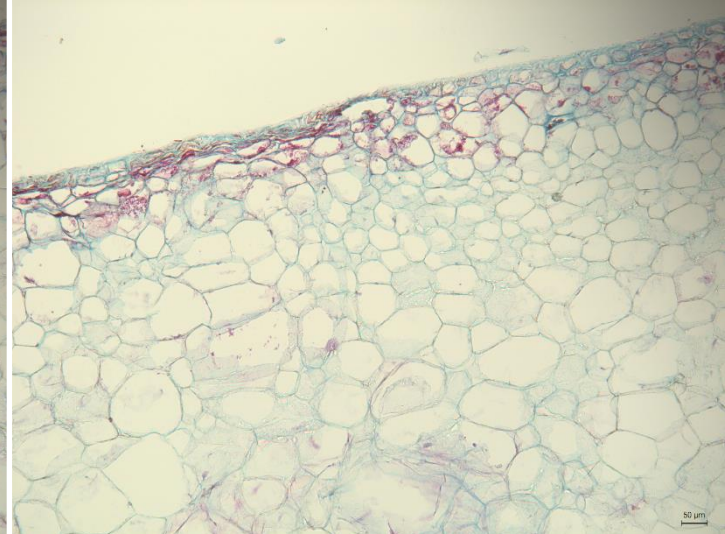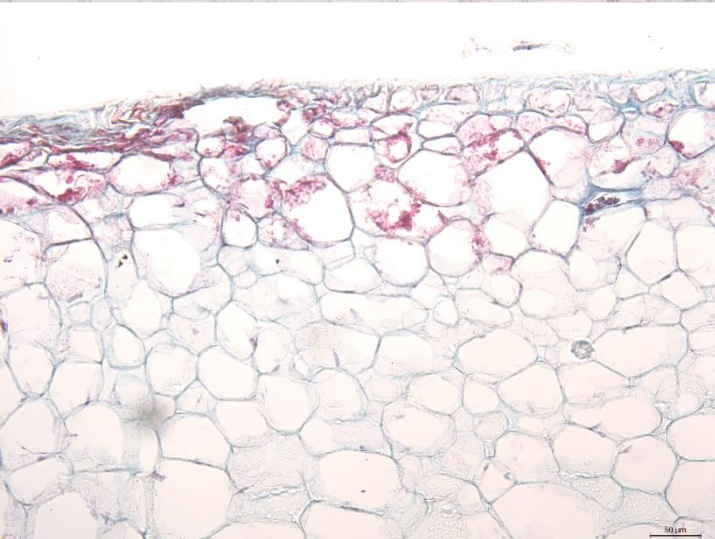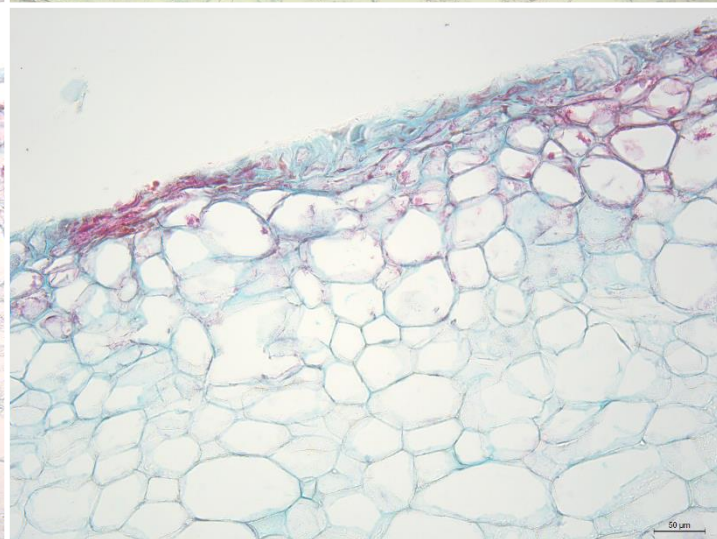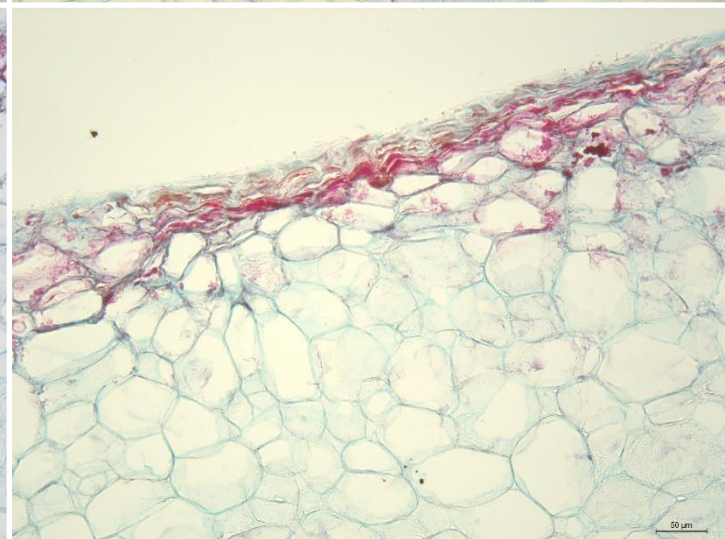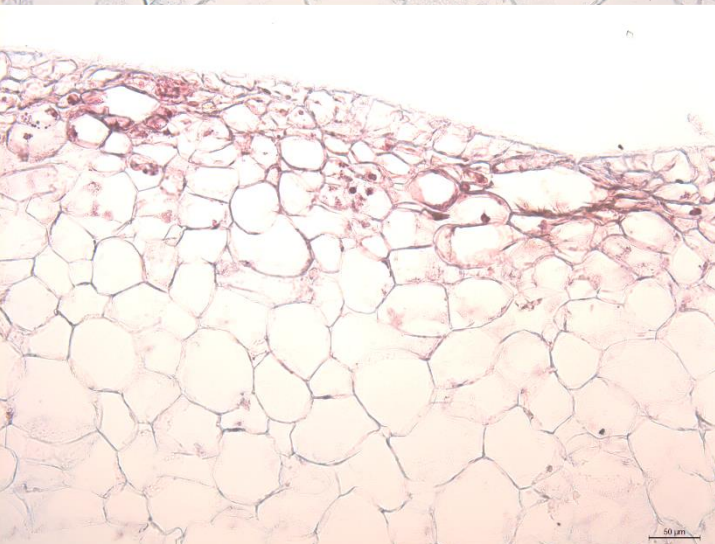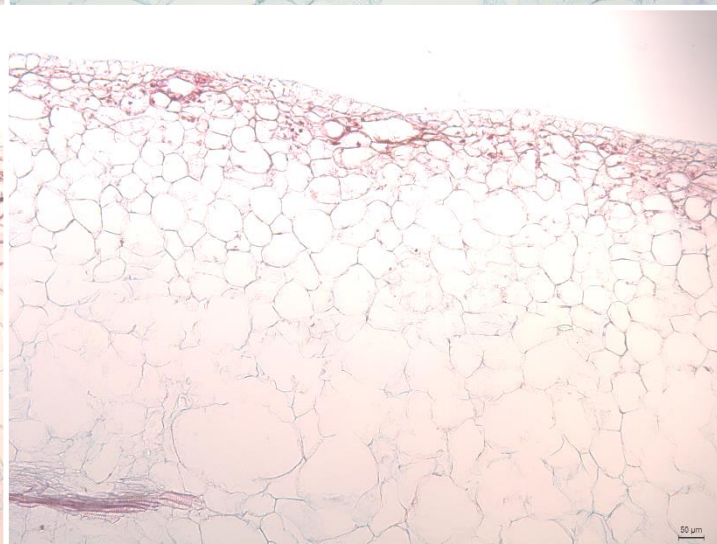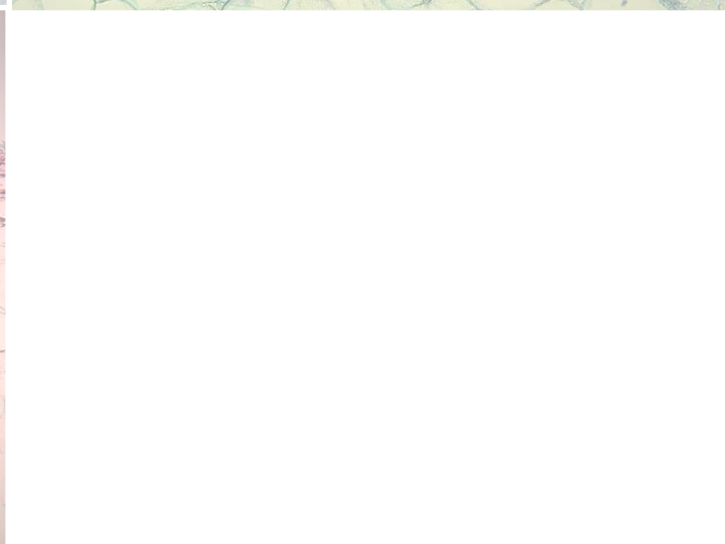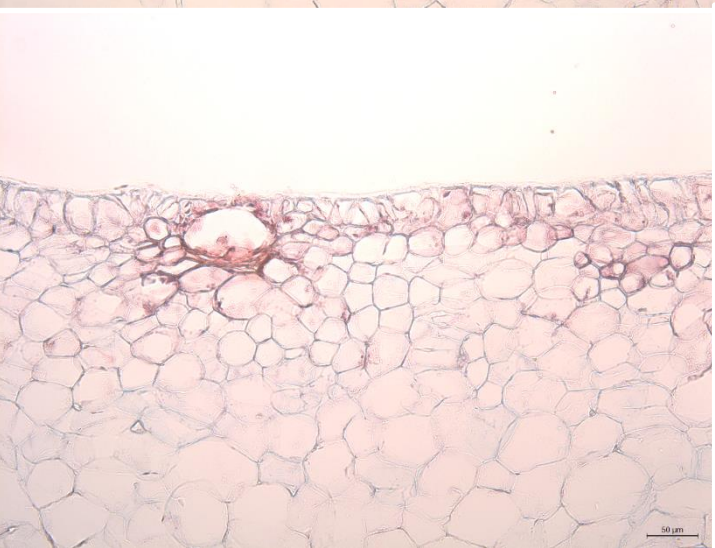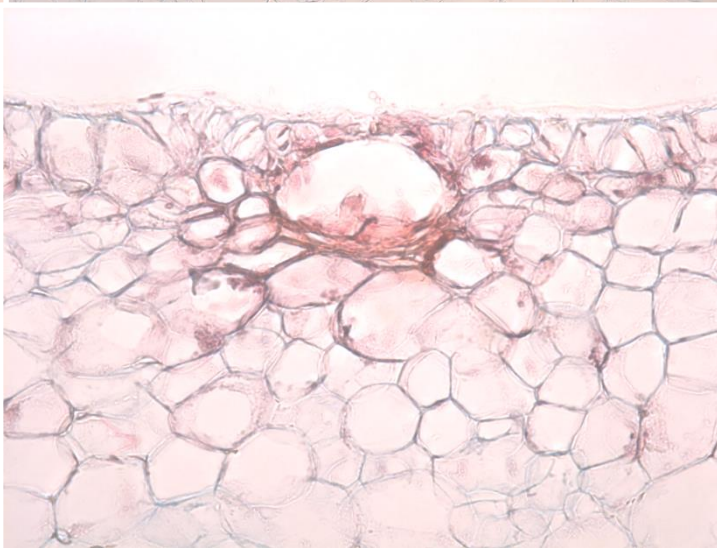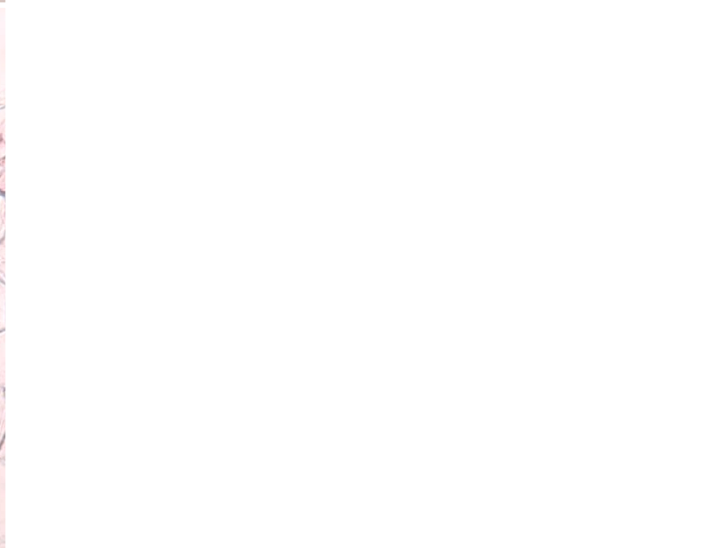

Supplement: S9 Fig — (PDF) [file pone.0160675.s009.pdf]

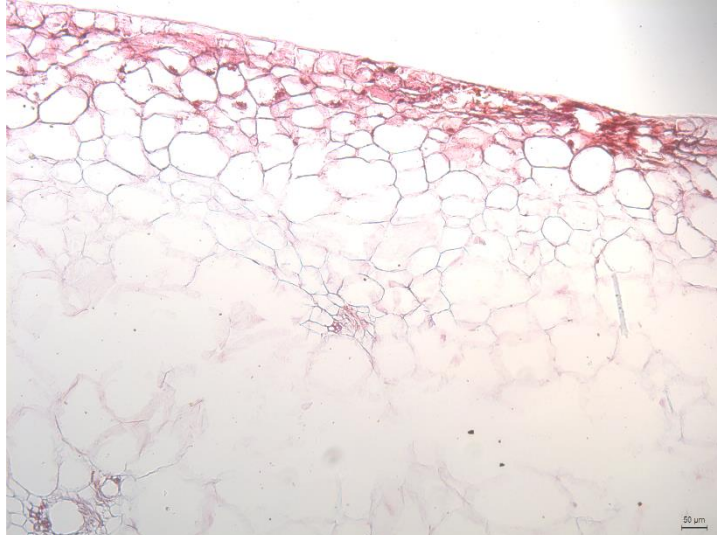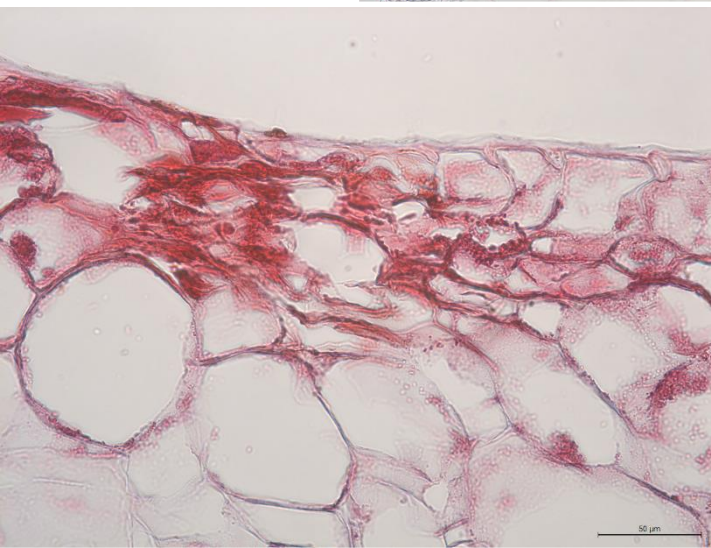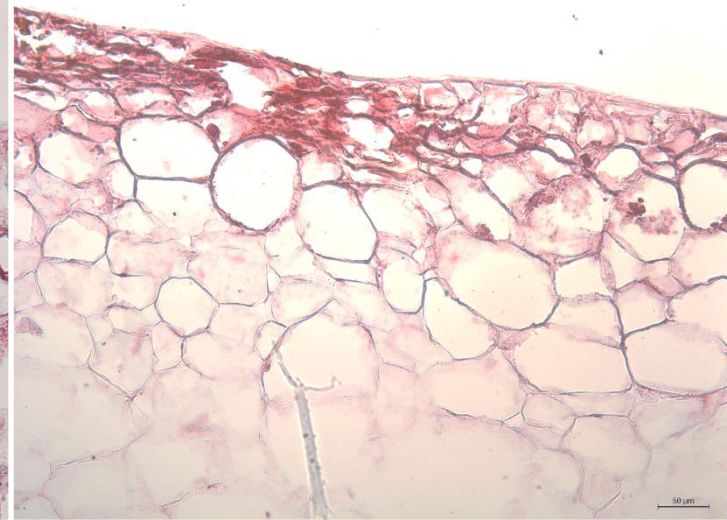

Supplement: S10 Fig — (PDF) [file pone.0160675.s010.pdf]

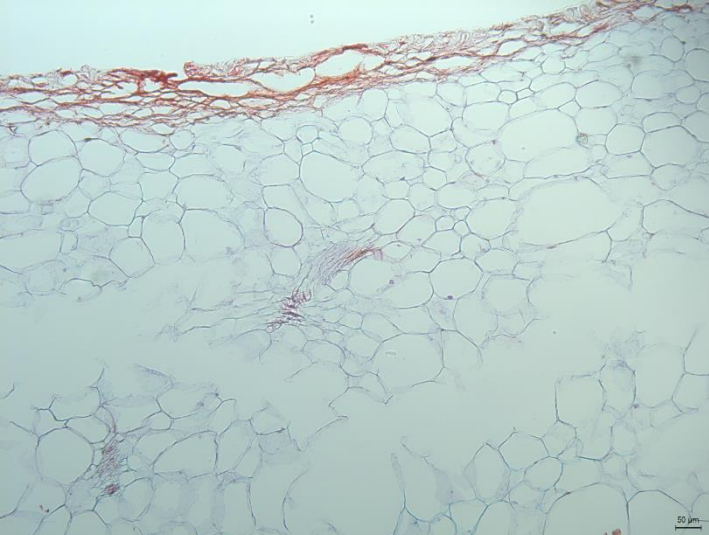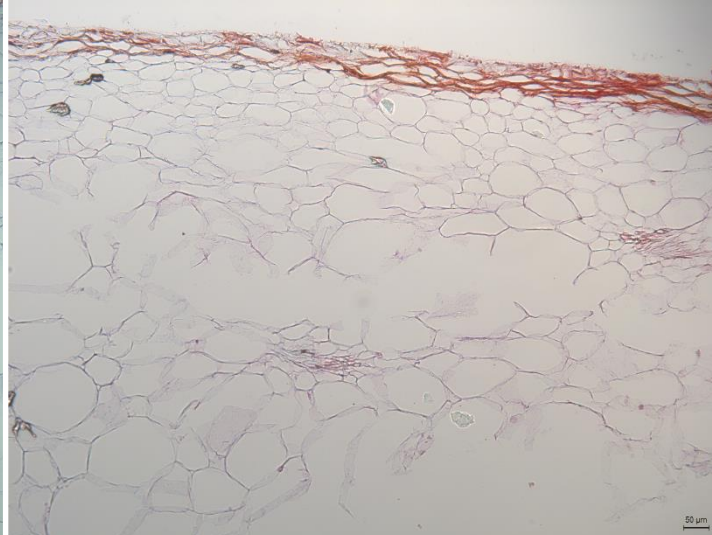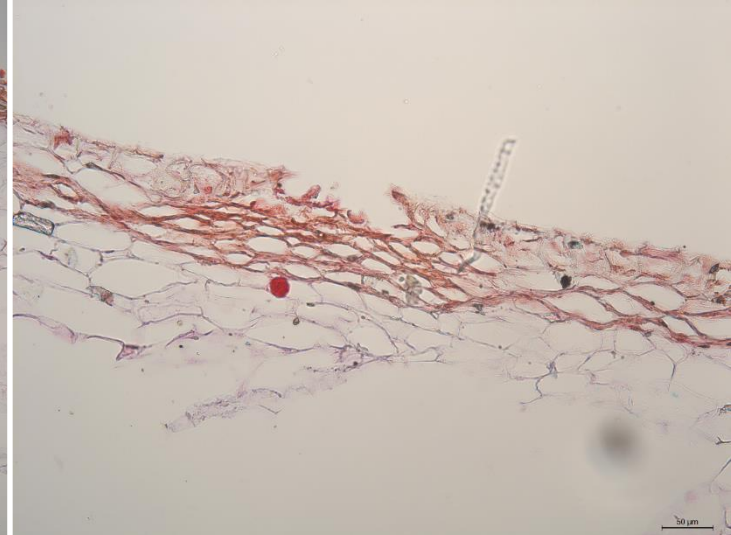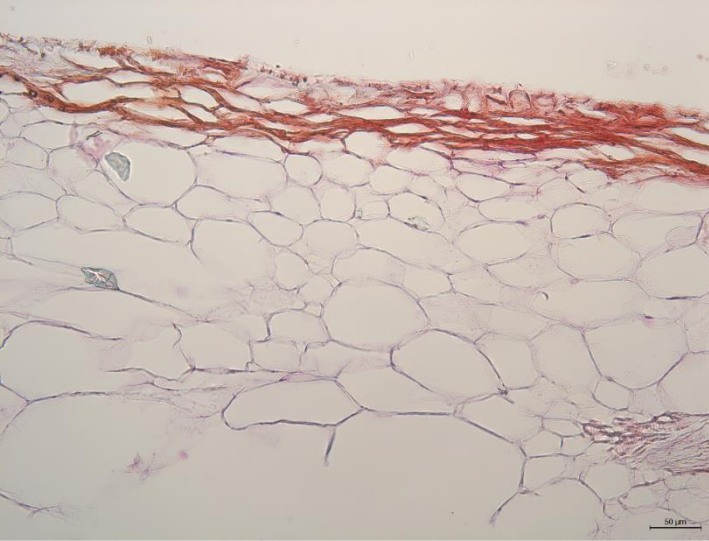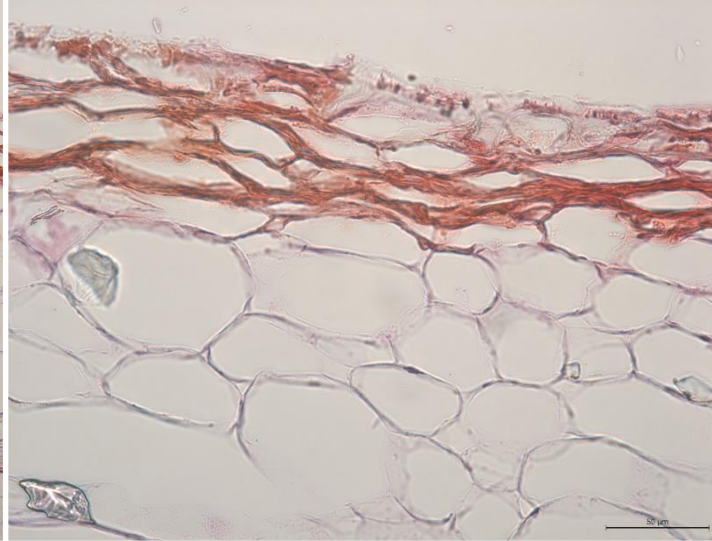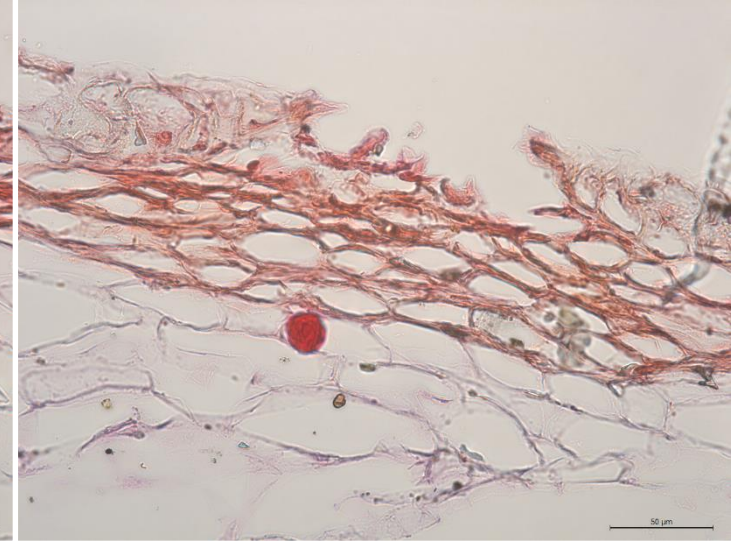

Supplement: S11 Fig — (PDF) [file pone.0160675.s011.pdf]

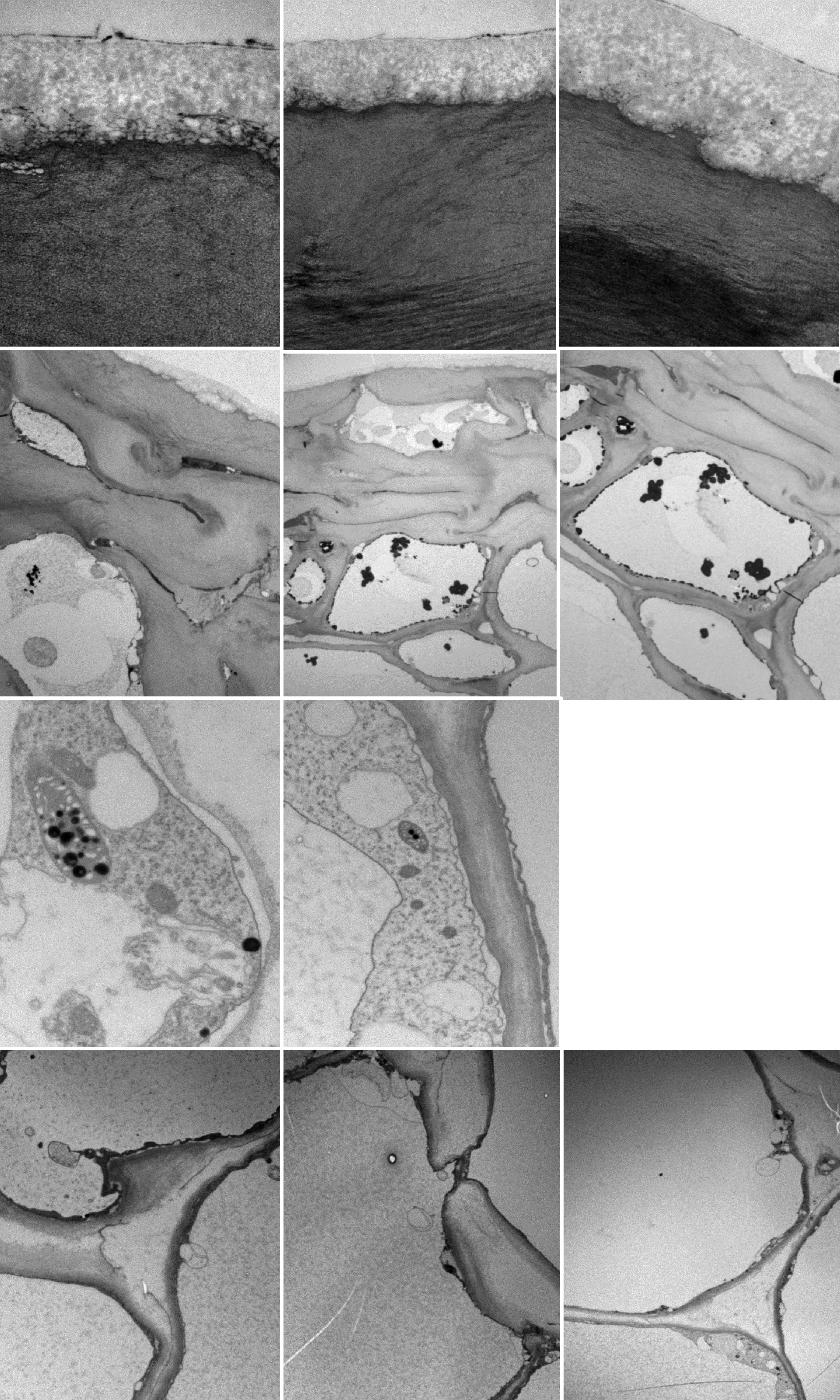

Supplement: S12 Fig — (TIF) [file pone.0160675.s012.tif]

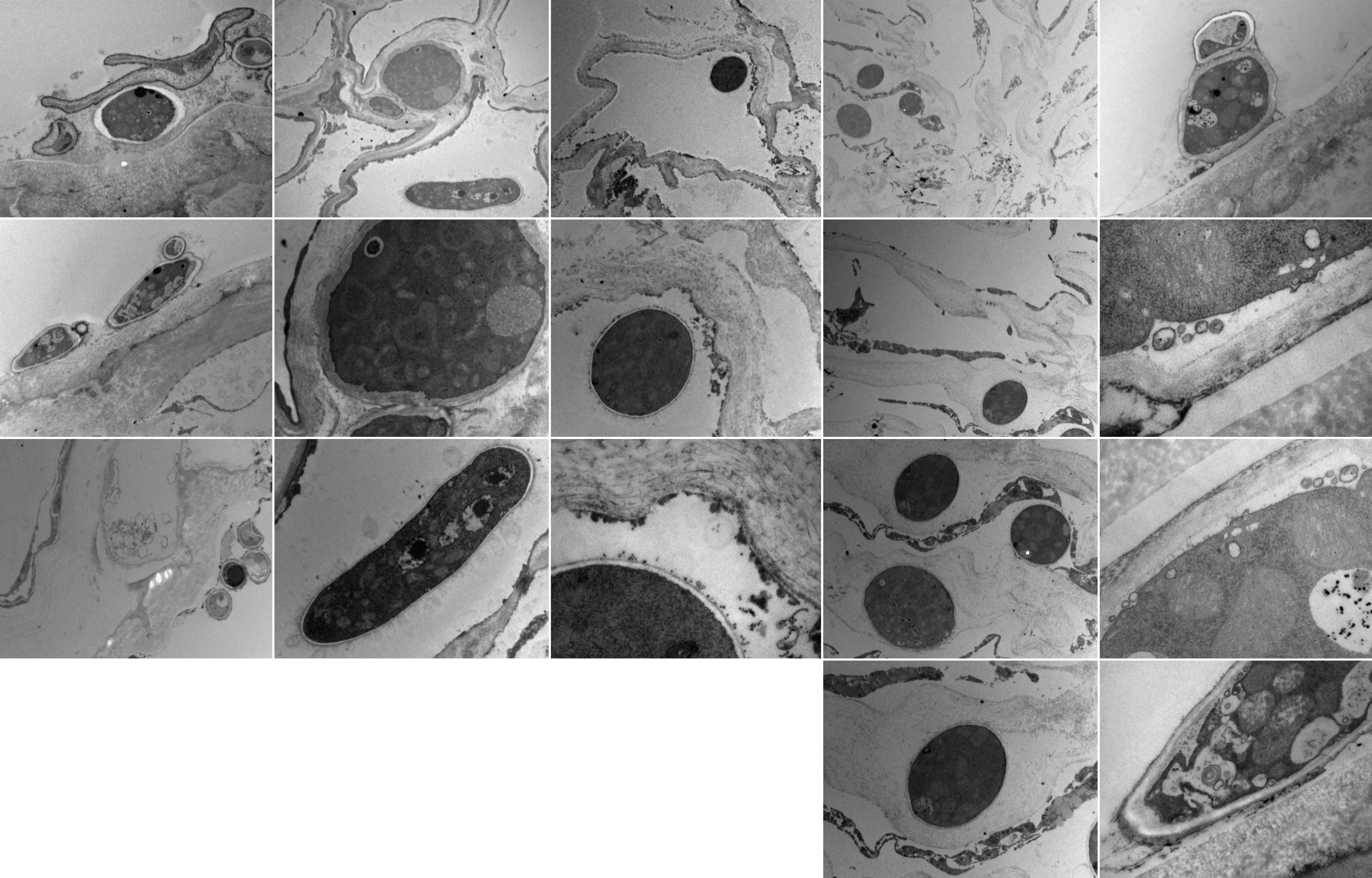

Supplement: S13 Fig — (TIF) [file pone.0160675.s013.tif]
